# Cigarette smoke exacerbates mouse allergic asthma through Smad proteins expressed in mast cells

**DOI:** 10.1186/1465-9921-12-49

**Published:** 2011-04-18

**Authors:** Dae Yong Kim, Eun Young Kwon, Gwan Ui Hong, Yun Song Lee, Seung-Hyo Lee, Jai Youl Ro

**Affiliations:** 1 Department of Pharmacology and Samsung Biomedical Research Institute, Sungkyunkwan University School of Medicine, Suwon 440-726, Korea; 2 Graduate School of Medical Science and Engineering, Korea Advanced Institute of Science and Technology, Daejeon, 305-701, Korea

## Abstract

**Background:**

Many studies have found that smoking reduces lung function, but the relationship between cigarette smoke and allergic asthma has not been clearly elucidated, particularly the role of mast cells. This study aimed to investigate the effects of smoke exposure on allergic asthma and its association with mast cells.

**Methods:**

BALB/c mice were sensitized and challenged by OVA to induce asthma, and bone marrow-derived mast cells (BMMCs) were stimulated with antigen/antibody reaction. Mice or BMMCs were exposed to cigarette smoke or CSE solution for 1 mo or 6 h, respectively. The recruitment of inflammatory cells into BAL fluid or lung tissues was determined by Diff-Quik or H&E staining, collagen deposition by Sircol assay, penh values by a whole-body plethysmography, co-localization of tryptase and Smad3 by immunohistochemistry, IgE and TGF-β level by ELISA, expressions of Smads proteins, activities of signaling molecules, or TGF-β mRNA by immunoblotting and RT-PCR.

**Results:**

Cigarette smoke enhanced OVA-specific IgE levels, penh values, recruitment of inflammatory cells including mast cells, expressions of smad family, TGF-β mRNA and proteins, and cytokines, phosphorylations of Smad2 and 3, and MAP kinases, co-localization of tryptase and Smad3, and collagen deposition more than those of BAL cells and lung tissues of OVA-induced allergic mice. CSE solution pretreatment enhanced expressions of TGF-β, Smad3, activities of MAP kinases, NF-κB/AP-1 or PAI-1 more than those of activated-BMMCs.

**Conclusions:**

The data suggest that smoke exposure enhances antigen-induced mast cell activation via TGF-β/Smad signaling pathways in mouse allergic asthma, and that it exacerbates airway inflammation and remodeling.

## Background

Cigarette smoke contains many toxic substances and a strong pro-inflammatory stimulus [1-3]. It is widely recognized as a significant risk factor for a number of diseases including emphysema, chronic obstructive pulmonary disease, cardiovascular disease, lung cancer and allergic diseases [1].

Effects of smoke on allergic airway inflammation in mice have reported both exacerbation [4-8] and attenuation [9-11], although these studies could not be directly compared due to differences in the various factors used, such as mouse strain, the routes and manners of allergen sensitization and smoke exposure. Smoke also enhanced airway hyperresponsiveness [12], but not IgE levels and eosinophils in mouse allergic model [12,13].

One particular factor which is involved in smoke-induced airway remodeling is transforming growth factor (TGF-β) [14]. The intracellular TGF-β-induced signaling pathway is mediated through the Smad pathway in inflammation in asthma [14-16]. TGF-β-producing T cells can suppress airway inflammation and hyperresponsiveness induced by Th2 effector cells in a murine allergic airway model [17,18]. However, it was recently shown that TGF-β/Smad2 signaling proteins were expressed in the majority of cells infiltrating into the airway in mouse models [19-22] and human asthma [19,23].

Mast cells are well-known as major effector cells for IgE-mediated allergic reactions such as asthma. Mast cells are activated by cross-linking of antigen-specific IgE bound to the high-affinity receptor (FcεRI) on their membranes. Activated mast cells secrete preformed mediators (histamine, tryptase, chymase, TNFα, and other proteins) as well as newly synthesized proinflammatory mediators such as PGD2, leukotrienes, cytokines, and chemokines [24]. These mediators contribute to airway inflammation and remodeling in allergic asthma [24,25]. TGF-β also acts as a negative regulator of mast cell function, TGF-β/Smad3-mediated signaling is essential for maximal cell growth in mast cells [26] and mast cell development via p38 kinase [27]. There are also controversial reports that cigarette smoke extract (CSE) solution contributes to the pathogenesis of emphysema and inflammation through proinflammatory chemokine production in mouse bone marrow-derived mast cells (BMMCs) [28], and that it suppresses allergic activation of in BMMCs [29].

Despite reports described above, cigarette smoke is controversial in development of allergic asthma, and a role of mast cells caused by smoke exposure has not been well understood, although they are related to allergic asthma. Therefore, we aimed to investigate whether cigarette smoke influences allergic/asthmatic reaction in mice, and whether mast cells are related to allergic reaction evoked by smoke exposure. We observed that cigarette smoke exposure exacerbates mouse airway inflammation and tissue remodeling via TGF-β/Smad proteins expressed by activated mast cells.

## Methods

### Reagents

Ovalbumin (OVA), alum (aluminum hydroxide, 2% Alhydrogel), methacholine, 3-(4,5-Dimethylthiazol-2-yl)-2,5-diphenyltetrazolium bromide (MTT), hematoxylin, eosin, PAS, van Gieson solution, DNP-BSA, anti-DNP IgE antibody, SB431542 were obtained from Sigma-Aldrich (St. Louis, MO); Cigarette (Marlboro) from Philip Morris (Lausanne, Switzerland); aprotinin, leupeptin from Roche (Baselm Switzerland); FITC-coupled goat anti-rabbit, Texas Red-coupled goat anti-mouse, lipofectamine from Invitrogen (Carlsbad, CA); Diff-Quick stain solution from International Reagents Corp. (Tokyo, Japan); May Grünwald-Giemsa solution, PD98059, SP600126, SB203580, PP2, piceaterol from Merck (Darmstadt, Germany); nitrocellulose membranes, chemiluminescent, [γ^32^P]ATP (specific activity, 3,000 Ci/mmol) from American Biosciences (Buckinghamshire, UK); peroxidase-conjugated goat anti-biotin antibody, mouse IgE from BD Biosciences (San Diego, CA); Sircol assay kit from Biocolor Ltd. (Carrickfergus, UK); primary-rabbit anti-Smad3, mouse anti-tryptase, Smad2, Smad 3, ERK, JNK, p38, PAI-1 from Santa Cruz Biotechnology (Santa Cruz, CA); HRP-conjugated rabbit anti-goat IgG from Zymed Laboratory Inc. (San Francisco, CA); TRIZOL from Molecular Research Center Inc. (Cincineti, OH); amfiRivert one-step RT-PCR kit from GenDEPOT (Barker, TX); anti-TGF-β, IL-4, -5, -6, TNF-α, biotinylated anti-TGF-β from BD Pharmingen (San Diego, CA); anti-IL-13 from R&D system (Minneapolis, MN); Silence Express Kit from Ambion Inc (Austin, TX); the oligonucleotide of NF-κB from Promega (Madison, WI); filter from Millipore (Bedford, MA).

### Sensitization and antigen challenge protocol

**Figure 1:**
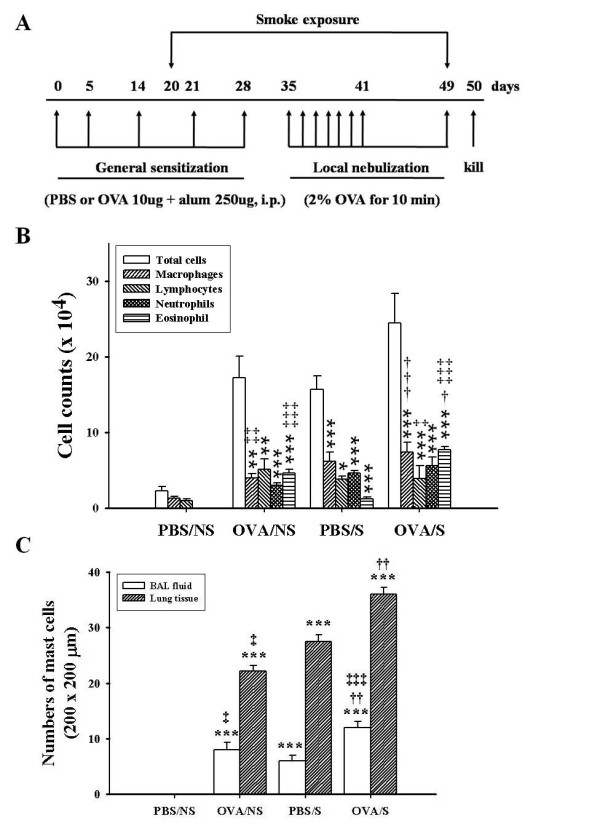
**Experimental protocol and effect of smoke exposure on the recruitment of inflammatory cells into BAL fluid or lung tissues of OVA-induced asthmatic mice**. BALB/c mice were sensitized with 10 μg OVA adsorbed in alum (250 μg) and nebulized with 2% OVA using nebulizer and exposed to smoke for 30 days (days 20 days 49) as described in "Materials and Methods" (A). PBS/NS, mice sensitized and nebulized by PBS without smoke exposure; OVA/NS, mice sensitized and nebulized by OVA without smoke exposure; PBS/S, mice sensitized and nebulized by PBS with smoke exposure; OVA/S, mice sensitized and nebulized by OVA with smoke exposure. General sensitization and local challenge were performed 10 min after final smoke exposure. BAL fluid collection was performed as described in "Materials and Methods". After Diff-Quik staining, differential cell counts were obtained by counting over 300 cells (B). Numbers of mast cells were determined in BAL cells collected by Cytospin or in lung tissues sectioned (3 μm) in 200 × 200 μm area under microscopy using May Grünwald-Giemsa staining (C). Data are shown as mean ± SEM for each group (n = 8). **, *P *< 0.01; ***, *P *< 0.001 versus PBS/NS mice. ^†^, *P *< 0.05; ^††^, *P *< 0.01; ^†††^, *P *< 0.001 versus OVA/NS mice. ^‡^, *P *< 0.05; ^‡‡^, *P *< 0.01; ^‡‡‡^, *P*<0.001 versus PBS/S mice.

Specific pathogen-free female BALB/c mice (Oriental Ltd, Seoul, Korea), 8 weeks of age, weighing approximately 20 g, were divided into four groups (8 mice/group). PBS/NS, mice sensitized and nebulized by PBS without smoke exposure; OVA/NS, mice sensitized and nebulized by OVA without smoke exposure; PBS/S, mice sensitized and nebulized by PBS with smoke exposure; OVA/S, mice sensitized and nebulized by OVA with smoke exposure. Hereafter, we used these group abbreviations to clarify the text. Mice were sensitized with 10 μg OVA (Grade V) adsorbed in 250 μg/200 μl of alum (aluminum hydroxide, 2% Alhydrogel) by i.p. injection on day 0, 5, 14, 21, and 28 (general sensitization) in all mice except control sensitized with PBS. One week after the final injection, mice were nebulized with 2% OVA for 7 consecutive days from day 35 to 41 (local challenge) using nebulizer (Mega Medical, Seoul, Korea), and then were again nebulized with 2% OVA on day 49. All controls were nebulized by PBS with or without smoke exposure at same times. All mice were sacrificed the following day (Figure 1A). General sensitization and local challenge were performed 10 min after final cigarette exposure.

### Cigarette smoke exposure in mice

The cigarette smoke exposure system used was a modification of one described previously [4]. Briefly, mice were subjected to whole-body mainstream cigarette smoke exposure produced by a cigarette in a Plexiglas chamber (16 × 25 × 16 cm) with an inlet for pressurized air (air: smoke = 3:1), connected to a smoking machine designed by Smoking Tester (Threeshine Com, Daejeon, Korea). Mice were exposed from day 20 to day 49 (30 days, subacute exposure model) according to the injection schedule of experimental protocol: on the first day (day 20), the smoke of a cigarette was administered for 10 min, and on the second day the smoke of two cigarettes was administered. The amount of cigarette smoke was gradually increased daily by one cigarette per day for five days. The interval between smoke exposures was 15 min. After the fifth day, the animals were exposed to five cigarettes/day from day 25 to day 49. We chose Marlboro cigarette for smoke exposure because total particulate matter (TPM, 56.7 ± 5.0 × 10^-3^/ml acridine orange units) of Marlboro cigarette is similar to TPM values of Kentucky Reference 2R4F (63.1 ± 4.6 × 10^-3^/ml acridine orange units) [30].

All animals were housed in accordance with guidelines from the Association for Assessment and Accreditation of Laboratory Animal Care (AAALAC), and all protocols were approved by the Institutional Review Board and conducted in the Laboratory Animal Research Center of Sungkyunkwan University.

### Measurement of OVA-specific IgE

Blood was collected by cardiac puncture and analyzed for OVA-specific IgE by ELISA [31]. Briefly, microplates coated with rat anti-mouse IgE were treated with mouse sera followed by biotinylated OVA. Reactions were read using an ELISA plate reader at 415 nm (see additional file for details).

### BAL fluid

BAL fluid was collected, stained and counted using methods previously described [31]. Numbers of infiltrated inflammatory cells or mast cells stained with Diff-Quick and May Grünwald-Giemsa [32], respectively, were quantified under microscopy (see additional file for details).

### Lung histology

After BAL had been performed, lungs tissue sections embedded in paraffin (3 μm) were stained with hematoxylin and eosin (H & E) for general morphology, with periodic acid-Schiff (PAS) for identification of goblet cells [31], with van Gieson for collagen deposition [33] and with May Grünwald-Giemsa for mast cells [32] (see additional file for details). Collagen amounts were assessed by the Sircol collagen assay according to the manufacturer's instruction (see additional file for details) [34].

The mean linear intercept (Lm) was determined by Lee et al. method [S1] (see additional file for details).

### Measurement of airway hyperresponsiveness

Airway responsiveness was measured in mice 24 h after the last challenge in conscious, spontaneously breathing mice using a whole-body plethysmography system (All Medicus Co., Seoul, Korea), as described previously [9,12]. Briefly, mice were placed in chamber and allowed to settle for 5 ~ 10 min, and the chamber-pressure-time wave was continuously measured via a transducer connected to a computer data-acquisition system. After baseline Penh reading for over a 5 min, mice were serially exposed to increasing concentrations of nebulized MCh (0, 3.125, 6.25, 12.5, 25 and 50 mg/ml) (Sigma-Aldrich, St. Louis, MO) in PBS for 2 min by inhalation. Penh values, which are measured as changes in enhanced pause, tidal volume (Vt), and breading frequency (breaths/minute) for the first 2 min after the end of MCh nebulization were averaged and used to compare responses between smoke exposure groups. Minute volumes were calculated by multiplying the Vt and breathing frequency.

### Immunohistochemistry (IHC)

Sections (μm) prepared in "Lung histology" were incubated with primary rabbit anti-Smad3 or mouse anti-tryptase, FITC-coupled goat anti-rabbit and Texas Red-coupled goat anti-mouse, and examined under Confocal microscopy (LSM 5 Exciter, Carl Zeiss, Oberkochen, Germany) [35]. The degree of IHC color (yellow) developed by co-localization was quantified by intensity in 100 × 100 μm areas under microscopy (5 areas/each slide × 8 mice/each group = 40 areas), and then mean ± SEM for 40 areas was presented by histogram (see additional file for details). Right lungs were removed and stored at -70°C for measurements of mRNA and protein expression.

### Preparation of the cigarette smoke extracts (CSE)

Water-soluble extract of cigarette smoke was prepared using the following method. Briefly, mainstream smoke from two commercial cigarettes was drawn through 7 ml RPMI-1640 media by application of a vacuum. Optical density (O.D) of cigarette smoke extract (CSE) solution was usually 2.0 ~ 2.4 at 340 nm when it was measured by spectrophotometer. This solution was filtered through a 0.22 μm pore size filter to remove bacteria and large particles. The CSE solution was freshly prepared before each experiment, and used within 10 min of preparation. In order to confirm cell viability in CSE solution, an MTT assay was performed (Additional file 1, Figure S1). The CSE solution was used in the stimulation of BMMCs at final concentration of 0.1, 0.5, and 1.0% [36]. Optimal concentration and time of CSE solution for BMMCs stimulation were 1.0% and 6 h, respectively, in preliminary experiments.

### Culture and activation of Bone marrow-derived mast cells (BMMCs)

Bone marrow cells flushed from femurs and tibias of BALB/c mice (female, 8 wk old) were cultured in RPMI-1640 and 50% WEHI-3B conditioned media for 5 wk and confirmed as described previously [32] (see additional file for details).

BMMCs (1 × 10^6 ^cells) were sensitized with anti-DNP IgE antibody (0.1 μg/ml) overnight at 37°C. The cells were washed and then challenged with 1.0 ng/ml DNP-HSA for time periods indicated (optimal time, 6 h) at 37°C in Tyrode's buffer [32]. CSE solution was treated for 6 h in DNP-HSA stimulation. MAP kinase inhibitors (50 μM PD98059 for ERK, 10 μM SP600125 for JNK, and 10 μM SB203580 for p38 kinase) or TGF-β receptor kinase inhibitor (10 μM SB431542) were added 30 min before DNP-HSA stimulation. Lyn and Syk kinase inhibitors (10 μM PP2 and 25 μM piceaterol, respectively) were added 10 min before DNP-HSA stimulation. In all experiments, optimal time and concentration were first determined in preliminary experiments.

### Immunoblotting for proteins

Immnunoblotting was conducted using a method previously described [31]. Briefly, BAL cells (1 × 10^6 ^cells), lung tissues (50 mg) or BMMCs (1 × 10^6 ^cells) were homogenized in lysis buffer, and then each protein was performed using immunoblotting (see additional file for details).

### Smad3 siRNA transfection

Smad3 siRNA-expressing vectors were generated using the Silencer Express Kit. Sense hairpin siRNA (ACA CTA CAC AAA TGT TCC ACT GGG CTG AGA ACC GGT GTT TCG TCC TTT CCA CAA G) and anti-sense hairpin siRNA (CGG CGA AGC TTT TTC CAA AAA ATT CTC AGC CCA GTG GAA CAC TAC ACA AAT G) template oligonucleotide, specific to Smad3 mRNA, were used [37].

Transfection was carried out according to the manufacture's method. Briefly, 1 μg vector that expresses Smad3 siRNA or control siRNA was incubated with 50 μl of serum free media for 5 min (Solution A), and 2 μl lipofectamine 2000 incubated with serum free media for 5 min (Solution B). Solution A was mixed with solution B, and incubated for 20 min. After incubation, BMMCs were added to the mixer. Transfected-BMMCs were activated with anti-DNP IgE antibody (0.1 μg/ml) and DNP-HSA (1.0 ng/ml) as methods described previously [32], and then the activities of MAP kinases, NF-κB, PAI-1 and expressions of cytokines were measured using western blot and RT-PCR, respectively.

### Reverse transcriptase-polymerase chain reaction (RT-PCR)

Total cellular RNA was isolated from BAL cells (1 × 10^6 ^cells), lung tissues (50 mg) or BMMCs (1 × 10^6 ^cells) using Trizol reagent. RT-PCR was performed in a final volume of 50 μl using an amfiRivert one-step RT-PCR kit in an automated thermal cycler (BIOER Technology, Hangzhou, China). PCR assays were performed for 35 cycles. PCR products were analyzed using 1.0% agarose gel containing ethidium bromide (EtBr) [31] (see additional file for details).

### ELISA

TGF-β, IL-4, IL-5, IL-13, TNF-α, and TGF-β (total and active forms) levels in lung tissue homogenates (1 mg/500 μl PBS) or in supernatants of activated BMMCs (1 × 10^6 ^cells) were determined by ELISA (see additional file for details).

### Electrophoretic mobility shift assay (EMSA)

Nuclear extracts were prepared from BMMCs (1 × 10^6 ^cells) and quantified using Bradford's method, and then electromobility shift assay were performed using the method previously described [31] (see additional file for details).

### Statistical analysis

Experimental data are presented as mean ± SEM. ANOVA was used for statistical analysis. An analysis of significance between each control group and experimental group was carried out with the SPSS statistic program ver.12 (SPSS Inc., Chicago, IL). *P *values < 0.05 were regarded as significant. However, the symbols comparing to OVA/S mice and OVA/NS mice or PBS/S mice was not indicated in some figures to avoid complexity. The relationship between OVA/NS and OVA/S mice was determined by linear regression analysis. The r^2 ^was used as a measure of how well the data fit the regression line. The densitometry analysis of immunoblots, PCR and EMSA was performed with Quantity One version 4.6.3 (BIO-RAD, Hercules, CA), and loading control used for bad images was not shown in all data. Histogram for densitometry analysis was indicated by mean ± SEM (n = 4) obtained from four independent experiments.

## Results

### Inflammatory cell recruitment into BAL fluid or lung tissues

We observed that OVA/S mice (24.5 ± 3.90 × 10^4 ^cells) enhanced recruitment of inflammatory cells (i.e., macrophages, neutrophils, and eosinophils, especially macrophage levels), compared to OVA/NS mice (17.2 ± 2.85 × 10^4 ^cells). PBS/S mice (15.7 ± 1.80 × 10^4 ^cells) also showed enhanced inflammatory cells versus the PBS/NS mice (2.3 ± 0.51 × 10^4 ^cells) (Figure 1B). The total cells (r^2 ^= 0.181, *P *= 0.014) and each inflammatory cells (Additional file 1, Figure S2) in BAL fluid of OVA/S mice were correlated with those of OVA/NS mice.

The numbers of mast cells were also elevated in OVA/S mice (12 ± 1.1 cells) by 50% compared to OVA/NS mice (8 ± 1.3 cells) in 200 × 200 μm areas under microscopy. PBS/S mice had elevated numbers of mast cells in BAL fluid, compared to PBS/NS mice (6 ± 1.1 cells versus 0 ± 0.0 cells) (Figure 1C).

In lung tissues, the numbers of mast cells were elevated in OVA/S mice (36 ± 1.2 cells) by 63.6%, compared to OVA/NS mice (22 ± 1.0 cells) or PBS/S mice (28 ± 1.2 cells) in 200 × 200 μm areas under microscopy (PBS/NS mice, 0 ± 0.0 cells) (Figure 1C). The increased mast cells in BAL fluid and lung tissues of OVA/S mice were positively correlated with those of OVA/NS mice (r^2 ^= 0.578, *P *= 0.029 for BAL cells; r^2 ^= 0.679, *P *= 0.044 for lung tissues) (Additional file 1, Figure S2).

### Histopathologic changes in lung tissues

**Figure 2:**
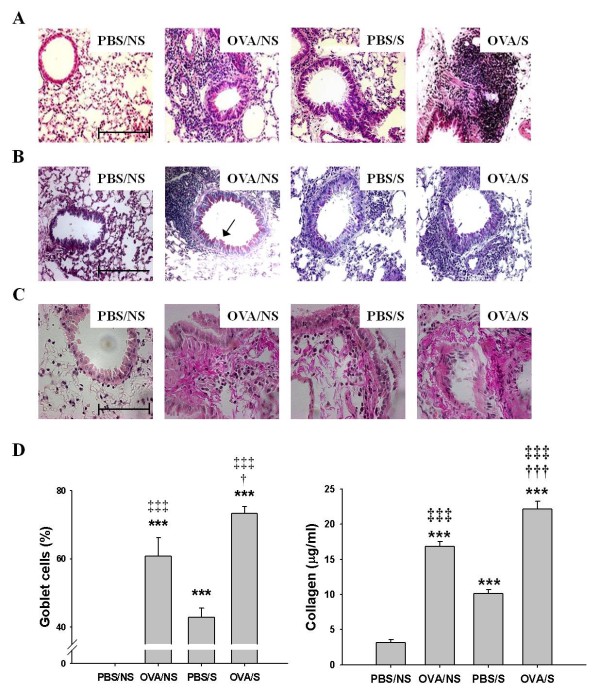
**Effects of smoke exposure on histopathological changes, goblet cell hyperplasia or collagen deposition in lung tissues of OVA-induced asthmatic mice**. Experimental conditions and group symbols used were described in Figure 1. Lung tissues were fixed with 4% paraformaldehyde, sectioned, and stained with hematoxilin and eosin (H & E) for general morphology (A) or periodic acid-Schiff (PAS) for goblet cell hyperplasia (B), and van Gieson (pink color) for collagen deposition (C) (magnification: × 200) and collagen amounts (D, right panel). Histogram indicates percentages in the numbers of PAS-positive goblet cells/total cells in 10 sites of 100 × 100 μm areas under microscopy (D, left panel). Bars in control (PBS/NS) image indicate 50 μm. Arrows in OVA/NS of B panel indicate goblet cells (pink color). Collagen amounts were assessed by the Sircol collagen assay. Data are shown as mean ± SEM for each group (n = 8). ***, *P *< 0.001 versus PBS/NS mice. ^†^, *P *< 0.05; ^†††^, *P*<0.001 versus OVA/NS mice. ^‡^, *P *< 0.05; ^‡‡‡^, *P *< 0.001 versus PBS/S mice.

We further observed increases in inflammatory cell infiltration in peribronchial and perivascular areas, thickening of the airway epithelium in the OVA/S mice, compared to OVA/NS mice (Figure 2A). And, mean linear intercept (Lm) for emphysema was enhanced in the OVA/S mice (43.8 ± 1.22 μm), compared to OVA/NS mice (35.2 ± 0.69 μm) or PBS/S mice (36.7 ± 0.48 μm). The Lm value in PBS/NS was 31.5 ± 0.86 μm (Additional file 1, Figure S3). However, the LM value for OVA/NS mice was weakly enhanced compared to PBS/NS mice, but there were no statistical difference.

Airway tissue remodeling is characterized by the goblet cell hyperplasia, deposition of collagen and hypertrophy of airway smooth muscle. We found that OVA/S mice (12.8 ± 0.74 cells/17.4 ± 0.63 total cells = 73.4%) had enhanced goblet cell hyperplasia (Figure 2B, 2D left panel) in the lumen of bronchi (10 sites of 100 × 100 μm areas under microscopy), compared to OVA/NS mice (8.6 ± 0.43 cells/14.2 ± 0.56 total cells = 60.7%) or PBS/S mice (6.5 ± 0.79 cells/15.2 ± 0.49 total cells = 42.8%). The increased goblet cells in lung tissues of OVA/S mice were positively correlated with those of OVA/NS mice (r^2 ^= 0.502, *P *= 0.022). And, OVA/S mice enhanced levels of collagen deposition (Pink color in Figure 2C). Collagen amounts were enhanced in OVA/S mice (22.2 ± 1.13 μg/ml) compared to that of OVA/NS mice (16.8 ± 0.72 μg/ml). And, in lung tissues of PBS/S mice, collagen amounts were enhanced, compared to that of PBS/NS mice (Figure 2D right panel).

### OVA-specific serum IgE levels

In order to more confirm the allergic asthma responses caused by smoke exposure, the effect of smoke on serum anti-OVA IgE level was also examined. Serum OVA-specific IgE levels were elevated in OVA/S mice (277.2 ± 7.58 ng/ml) more than that in OVA/NS mice (228.1 ± 5.34 ng/ml), through both had levels higher than in PBS/NS mice (0.5 ± 1.34 ng/ml) or PBS/S mice (0.6 ± 1.67 ng/ml) (Table 1).

### Airway hyperresponsiveness (AHR) to methacholine (MCh)

**Figure 3:**
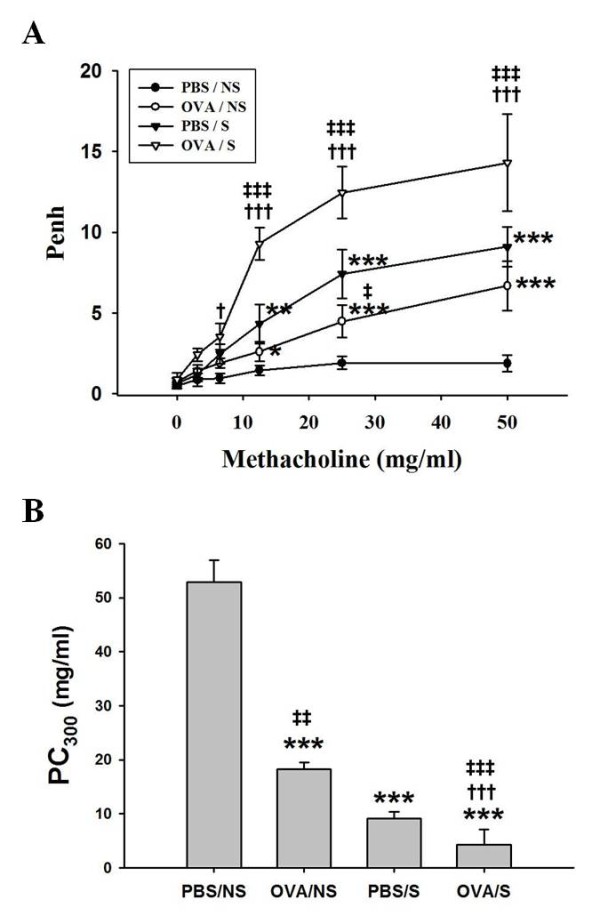
**The effects of smoke exposure on airway hyperresponsiveness in OVA-induced asthmatic mice**. Experimental conditions and group symbols used were described in Figure 1. Airway hyperresponsiveness was measured at 24 h after last challenge using a whole-body plethysmography as described in "Materials and Methods". Airway responsiveness to aerosolized methacholine (MCh) was measured in conscious, spontaneously breathing mice. Mice were placed into the main chamber and were nebulized first with PBS, then with increasing doses (1.56 to 50 mg/ml) of MCh for 2 min for each nebulization. Readings of breathing parameters were taken for 2 min after each nebulization during which Penh values were determined (A). The PC300 represents the dose of MCh which induces 300% increase in Penh (B). Data are expressed as mean ± S.E.M. (n = 8). *, *P *< 0.05; **, *p *< 0.01; ***, *p *< 0.001 versus PBS/NS mice. ^†^, *P *< 0.05; ^†††^, *P*<0.001 versus OVA/NS mice. ^‡^, *P *< 0.05; ^‡‡^, *P *< 0.01; ^‡‡‡^, *P *< 0.001 versus PBS/S mice.

AHR was measured 24 h after final exposure. Baseline Penh values were not significantly affected by allergic challenge or smoking groups (from 0.51 ± 0.21 to 0.88 ± 0.39). Penh values OVA/NS mice had a significant increase in MCh dose-dependent manner (from 3.125 to 50 mg/ml). OVA/S mice enhanced Penh values more than those in OVA/NS or PBS/S mice (Figure 3A). PBS/S mice also enhanced Penh values more than that in PBS/NS mice, but did not show significant differences compared to in OVA/NS mice. PC300 values were decreased in OVA/NS mice (18.3 ± 1.15 mg/ml) compared to PBS/NS mice (52.8 ± 4.06 mg/ml), and they were decreased in OVA/S mice (4.3 ± 2.80 mg/ml) more than that in OVA/NS or PBS/S mice (9.1 ± 1.17 mg/ml) (Figure 3B). Smoking had effect on AHR in OVA-challenged mice.

Tidal volume (Vt) was weakly decreased with the functional residual volume increase after mouse airways were challenged with MCh. The Vt in OVA/NS mice was inversely reduced with the increasing dosage of MCh, and it was reduced in OVA/S mice more than that in OVA/NS mice which were decreased compared with PBS/NS mice (Additional file 1, Figure S3B). Breathing frequency was increased in OVA/S mice more than that in OVA/NS or PBS/S mice (Additional file 1, Figure S3C). Minute volume was weakly decreased in OVA/S mice compared to OVA/NS or PBS/S mice (data not shown).

### Expression and activity of TGF-β and Smad family proteins in BMMCs

**Figure 4:**
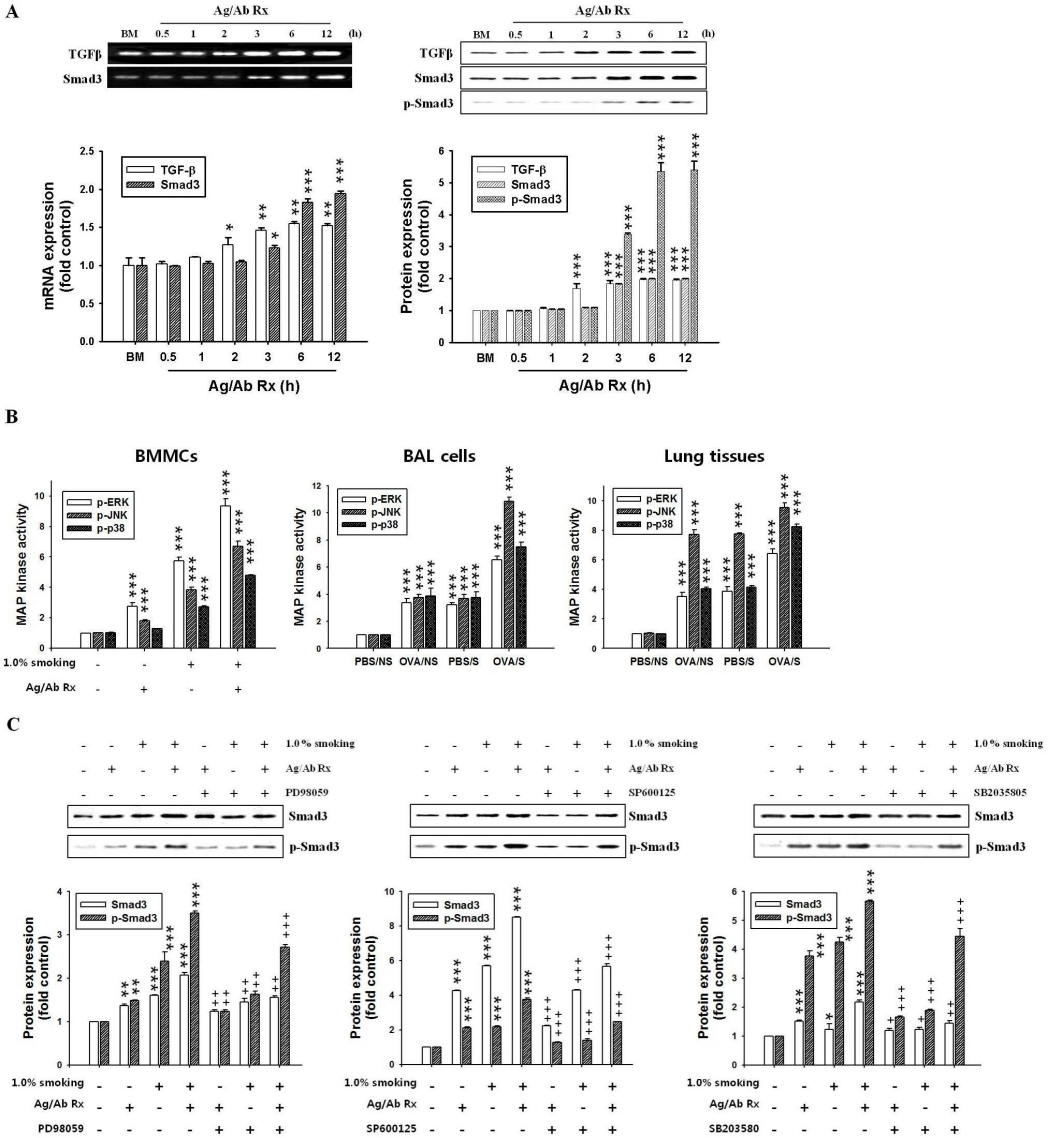
**Effects of smoke exposure or MAP kinase inhibitors on the TGF-β expression, or Smad3 activity in BMMCs activated with antigen/antibody reaction or in BAL cells and lung tissues of OVA-induced asthmatic mice**. BMMCs (1 × 10^6 ^cells) were sensitized and stimulated with 0.1 μg/ml anti-DNP IgE antibody and DNP-HSA (1.0 μg/ml) for 6 h, CSE solution (OD 2.0, 1.0%) was treated during DNP-HSA stimulation. mRNA expressions and activities were determined using RT-PCR and western blot as described in "Materials and Methods". Experimental conditions for BAL cells and lung tissues and group symbols used are described in Fig. 1. MAP kinase inhibitors (50 μM PD98059 for ERK, 10 μM SP600125 for JNK, and 10 μM SB203580 for p38 kinase) were added 30 min before DNP-HAS stimulation. Mean ± SEM (n = 4) for values measured by densitometry analysis was indicated by histogram. (A) Band images and histogram for time course in mRNA expression (left panel) and activity (right panel) of TGF-β and Smad3. (B) Histogram for activities of MAP kinases. (C) Band images and histogram for activities of Smad3 after pretreatment of MAP kinase inhibitors. Ag/Ab Rx, anti-DNP IgE antibody/DNP-HSA reaction; BM, BMMCs; -, +, without and with substances. *, *P *< 0.05; **, *P *< 0.01; ***, *P *< 0.001 versus control (BMMCs alone) or PBS/NS mice. ^+^, *P *< 0.05; ^++^, *P *< 0.01; ^+++^, *P *< 0.001 versus BMMCs without MAP kinase inhibitor. In Figure 4A and 4B, the symbols comparing to OVA/S mice and OVA/NS mice or PBS/S mice was not indicated to avoid complexity.

Enhancement of inflammatory cells (macrophages, neutrophils and eosinophils) was previously reported in OVA-induced allergic mice exposed with smoke by other [5,8,9] and our laboratories (Figure 1B and Figure 2A), but enhancement of mast cells was not reported yet. And, mast cells are involved in allergic inflammation and remodeling [25] and cigarette smoking is linked to the TGF-β/Smad signaling pathways in airway remodeling [14]. Therefore, we focused on mast cells, and we first examined the expressions of TGF-β/Smad proteins using primary cells (BMMCs) in vitro. In BMMCs activated with anti-DNP IgE/DNP-HSA reaction (refer to the activated-BMMCs), we observed higher mRNA expression of Smads throughout a time course (left panel) and activity (right panel) of Smads in the activated-BMMCs treated with CSE (refer to CSE-treated/activated-BMMCs), compared to the activated BMMCs (Addition file 1, Figure S4A). And, mRNA levels (left column) and protein expression (right column) of TGF-β were observed to reach a maximum at each 3 h, and mRNA expression and activity of Smad3 reached a maximum at each 6 h (Figure 4A).

In order to investigate the mechanism of action of smoke exposure on total and active TGF-β release and expression of all Smad proteins in BMMCs, we observed that CSE- treated/activated-BMMCs) released total and active TGF-β more than did only activated-BMMCs or only CSE-treated cells (Table 2 right column).

Furthermore, protein or mRNA expression and phosphorylation of Smad3 was inhibited by TGF-β receptor kinase inhibitor SB431542 (Addition file 1, Figure S4C), but not by Lyn or Syk kinase inhibitor, PP2 and pice (Addition file 1, Figure S4B).

### Activities of MAP kinases or transcription factors in BMMCs

MAP kinases phosphorylate Smad3 linker regions through TGF-β-induced activation in various cells [38,39]. In Figure 4B, the activities of MAP kinases were enhanced in CSE-treated/activated-BMMCs, compared to those of the activated-BMMCs (Figure 4B left panel). MAP kinase inhibitors blocked phosphorylation of Smad3 (Figure 4C) as well as phosphorylation of their respective MAP kinases (data not shown).

We observed the enhanced NF-κB and AP-1 DNA binding activity or expression of PAI-1 in nuclear extracts from CSE-treated/activated-BMMCs, compared to the activated-BMMCs. The enhancement in NF-κB and AP-1 or PAI-1 activity was reduced by inhibitors of MAP kinases (Addition file 1, Figure S5A,B). Expression patterns for transcription factors in lung tissues of OVA/S mice also showed similar results to those of BMMCs (data not shown).

### Effects of Smad3 siRNA on the activities of MAP kinases, transcription factors and cytokine expression in BMMCs

**Figure 5:**
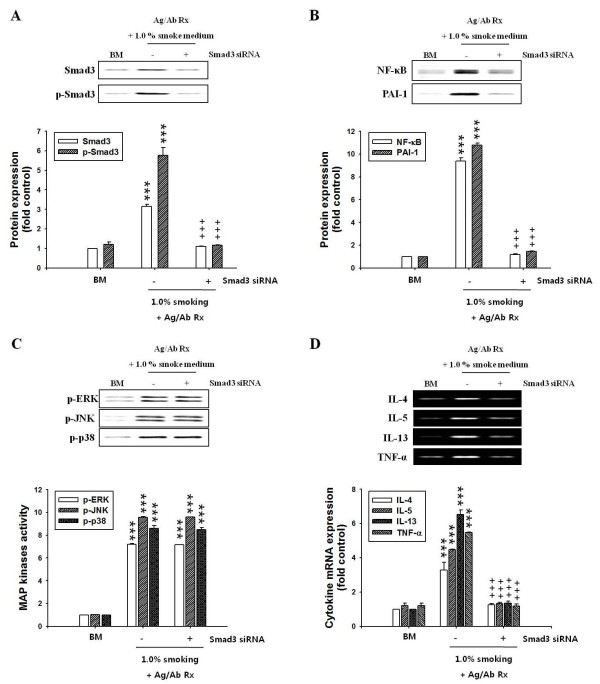
**Effects of Smad3 siRNA on activities of MAP kinases, NF-κB/PAI-1, and expression of cytokines in BMMCs activated with antigen/antibody reaction**. Smad3 siRNA-expressing vectors were generated in BMMCs using the Silencer Express Kit. Transfected BMMCs (1 × 10^6 ^cells) were activated with 0.1 μg/ml anti-DNP IgE antibody and DNP-HSA (1.0 μg/ml) for 6 h. CSE solution (OD 2.0, 1.0%) was treated during DNP-HAS stimulation, as described in "Materials and Methods". Expression or activity of Smad3, MAP kinases, transcription factors and cytokines were determined in extract protein and nuclear protein extracts prepared from BMMCs using western blot and RT-PCR, respectively. Mean ± SEM (n = 4) for values measured by densitometry analysis was indicated by histogram. (A) Band images and histogram for protein and activity of Smad3. (B) Band images and histogram for expressions of NF-κB and PAI-1. (C) Band images and histogram for activities of MAP kinases. (D) Band images and histogram for cytokine mRNA expressions. BM, BMMCs; Ag/Ab Rx, anti-DNP IgE antibody/DNP-HSA reaction; -, +, without and with Smad3 siRNA. ***, *P *< 0.001versus control (BMMCs alone). ^+++^, *P *< 0.001 versus CSE-treated/activated-BMMCs.

TGF-β signaling pathways include the TGF-β type I receptor (TβRI)/C-terminal phosphorylated Smad3 (pSmad3) pathway. Therefore, we examined whether Smad3 siRNA influenced the activities of MAP kinases and NF-κB/PAI-1 downstream of Smad3 in activated-BMMCs. We performed Samd3 siRNA in BMMCs' cells, and Smad3 siRNA did not enhance the activities of pSmad3 (Figure 5A) and NF-κB/PAI-1 in CSE-treated/activated-BMMCs (Figure 5B), but the activities of MAP kinases were enhanced regardless Smad3 siRNA (Figure 5C).

### mRNA and protein expressions of cytokine in BAL cells and lung tissues or BMMCs

Just as transcription factors NF-κB and AP-1 regulate the production of various inflammatory cytokines, we observed that mRNA of various cytokines (IL-1β, -4, -5, -6 -10, -13, TNF α and IFNγ) were enhanced in BAL cells and lung tissues of OVA/S mice or CSE- treated/activated-BMMCs versus those of OVA/NS mice or activated-BMMCs (Addition file 1, Figure S6). Each MAP kinase inhibitor significantly attenuated the increase in expression of cytokine mRNA (data not shown).

We also examined protein expression of some cytokines (IL-4, -5, -6, -13, TNFα) among various inflammatory cytokine mRNA (Addition file 1, Figure S6). These cytokine expressions were enhanced in lung tissues of OVA/S mice, compared to OVA/NS mice (Table 3). And, Smad3 siRNA transfection did not enhance expression of cytokine mRNA in CSE treated/activated-BMMCs (Figure 5D).

### Expression of TGF-β and Smad family members in BAL cells or lung tissues

**Figure 6:**
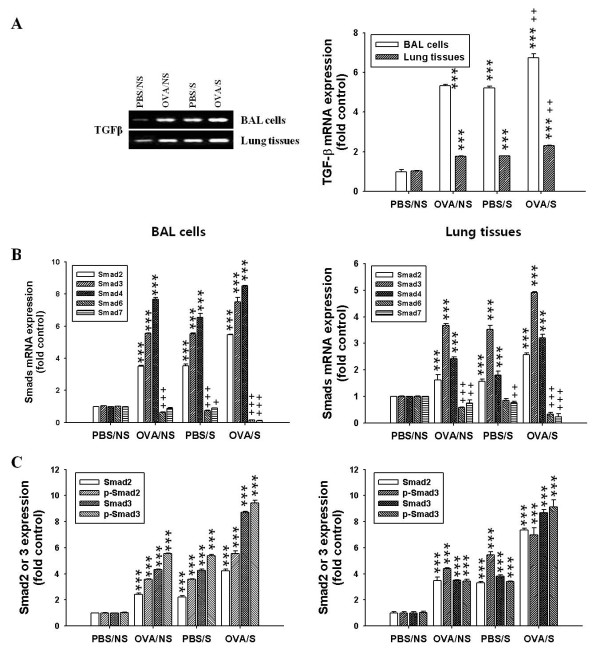
**Effects of smoke exposure on mRNA expressions of TGF-β and Smad family members in BAL cells or lung tissues of OVA-induced asthmatic mice**. Experimental conditions and group symbols used were described in Fig. 1. BAL cells or lung tissues were collected. mRNA expressions of TGFβ and Smads or Smad3 phosphorylation were analyzed by RT-PCR and western blot, respectively. Mean ± SEM (n = 4) for values measured by densitometry analysis was indicated by histogram. (A) Band images (left panel) and histogram (right panel) for TGF-β mRNA expressions. (B) Histogram for mRNA expressions of Smads. (C) Histogram for expressions and activities of Smad2/3. ***, *P *< 0.001versus PBS/NS mice. ^+++^, *P *< 0.001 versus OVA/NS or PBS/S mice. In Figure 5B and 5C, the symbols comparing to OVA/S mice and OVA/NS or PBS/S mice did not indicate to avoid complexity. The symbols comparing to OVA/S mice and OVA/NS mice or PBS/S mice was not indicated to avoid complexity.

Next, we further examined whether these phenomena evoked by BMMCs in vitro were caused in OVA-induced mice in vivo. We observed that expression of TGF-β mRNA (Figure 6A) was higher in BAL cells or lung tissues of OVA/S mice, compared to the levels in OVA/NS mice or PBS/S mice. Amounts of total and active TGF-β also were higher in lung tissues of OVA/S mice than those of OVA/NS mice or PBS/S mice (Table 2 left panel).

We next observed that mRNA expression of Smad family (Figure 6B) and phosphorylation of Smad2 and 3 (Figure 6C) induced by TGF-β signaling were more elevated in BAL cells or lung tissues of OVA/S mice, compared with those of OVA/NS mice. However, expression of inhibitory Smad6 and 7 mRNA was decreased in BAL cells and lung tissues of OVA/S mice, compared to those of OVA/NS mice (Figure 6B).

As shown in activated-BMMCs (Figure 4B left panel), the activities of MAP kinases were also enhanced in BAL cells (middle panel) and lung tissues (right panel) of OVA/S mice, compared to those of OVA/NS mice or PBS/N mice (Figure 4B).

### Co-localization of mast cell tryptase and Smad3 protein in lung tissues

**Figure 7:**
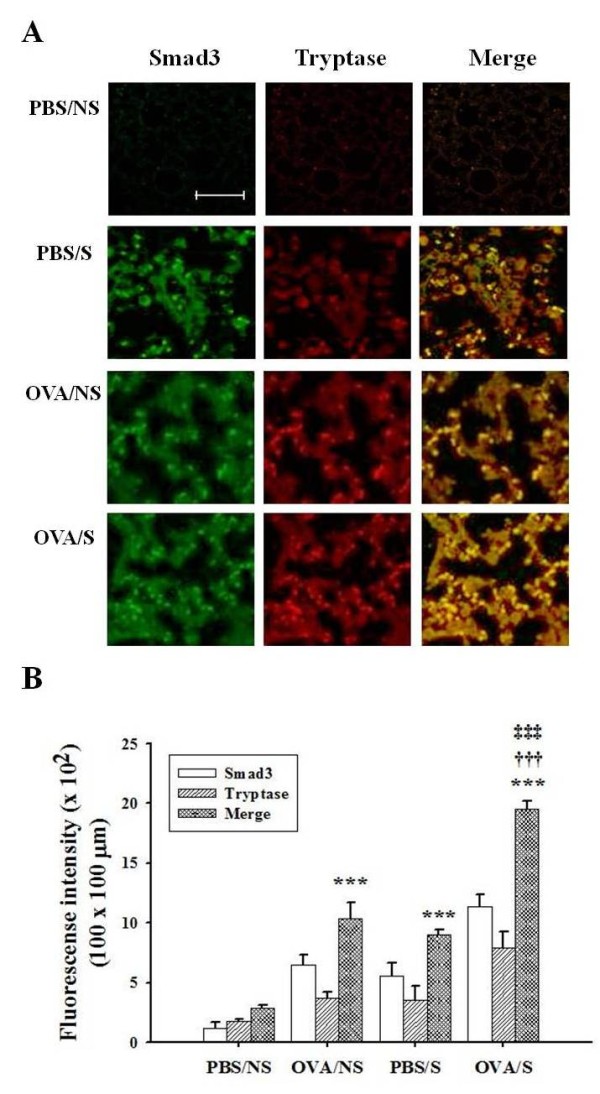
**Effects of smoke exposure on co-localization of mast cell tryptase and Smad3 in lung tissues of OVA-induced asthmatic mice**. Experimental conditions and group symbols used were described in Fig. 1. Lung tissues were fixed with 4% paraformaldehyde, sectioned and immunostained as described in "Materials and Methods". Co-localization was examined by immunohistochemistry (A). The degree of IHC color (yellow) developed by co-localization was quantified by intensity in 100 × 100 μm areas under microscopy (5 areas/each slide × 8 mice/each group = 40 areas), and then mean ± SEM for 40 areas was presented by histogram (B). ***, *P*<0.001 versus PBS/NS mice. ^†††^, *P *< 0.001 versus OVA/NS mice. ^‡‡‡^, P < 0.001 versus PBS/S mice. Bar in control (PBS/NS) image indicates 50 μm.

In order to investigate whether smoke-induced expression of Smad proteins was occurring in mast cells, we examined co-localization of tryptase contained in mast cell granules and Smad3 protein in lung tissues using double staining. As shown in Figure 7A, lung tissues in OVA/S mice enhanced the co-localization (yellow color) of tryptase and Smad3 protein, compared with PBS/S or OVA/NS mice. The intensity of co-localized cells in 100 × 100 μm areas under microscopy (5 areas/each slide × 8 mice/each group = 40 areas) was presented as histogram (Figure 7B).

## Discussion

It has been reported that cigarette smoke may promote [4-8] or actually suppress [9-11] allergic inflammation. However, the mechanisms involved have not been elucidated clearly. We observed that cigarette smoke exacerbates the development of allergic asthma by mast cell activation through TGF-β/Smad signaling.

Cigarette smoke elevates the numbers of prominent inflammatory cells such as macrophages and neutrophils or goblet cells, and it enhances release of inflammatory cytokines [3,4,40]. As smoke has adjuvant property [5,7,8], these cells play important roles in the exacerbation of asthmatic airway inflammation [4,5]. And, IL-4 plays an important role in B cell growth, differentiation, and secretion of IgE and IL-13 [40,41]. Our data showing that cigarette smoke augmented numbers of their inflammatory cells, the mRNA and protein levels of IL-4 and IL-13 in BAL cells and lung tissues, or in BMMCs and OVA-specific IgE levels and Penh values, are in agreement with the previous reports that smoking enhanced inflammatory cells, OVA-specific IgE production, and cytokines.

Mast cells have been implicated in airway inflammation and remodeling [24,26], but long-term smoking (4 mo) did not show enhanced numbers of mast cells in OVA-induced lung tissues in vivo [13]. In contrast to these findings, we observed that smoke elevated the numbers of mast cells in BAL cells and lung tissues of OVA/S mice, although both studies differed in various factors such as smoke exposed periods and allergen challenge.

Mast cells in tongue and esophageal submucosa express TGF-β [42]. TGF-β attenuates release of mediators and *de novo *kit receptor expression through Smad pathway in human skin mast cells [43]. However, TGF-β is increased at the sites of allergic inflammation [23], may contribute to tissue fibrosis and airway remodeling [25,44], and is involved in smoke-induced airway remodeling [14]. TGF-β overexpression is associated with initial events occurring during the emphysematous process [45]. Furthermore, production of chemokines increased in BMMCs activation by smoke exposure contributes to the pathogenesis of emphysema and a local inflammation [28]. Our data demonstrated that OVA/S mice enhance emphysema and expressions of TGF-β and Smad proteins in vivo and co-localization of mast cells and Smad3 protein in lung tissues. We also infer that OVA/NS mice may induce emphysema due to the expression of TGF-β and Smad proteins in vivo, although a degree of emphysema development is weaker than that of OVA/S mice. This is the first report of TGF-β/Smad pathways being involved in smoke-exposed/OVA-induced activation of mast cells.

**Figure 8:**
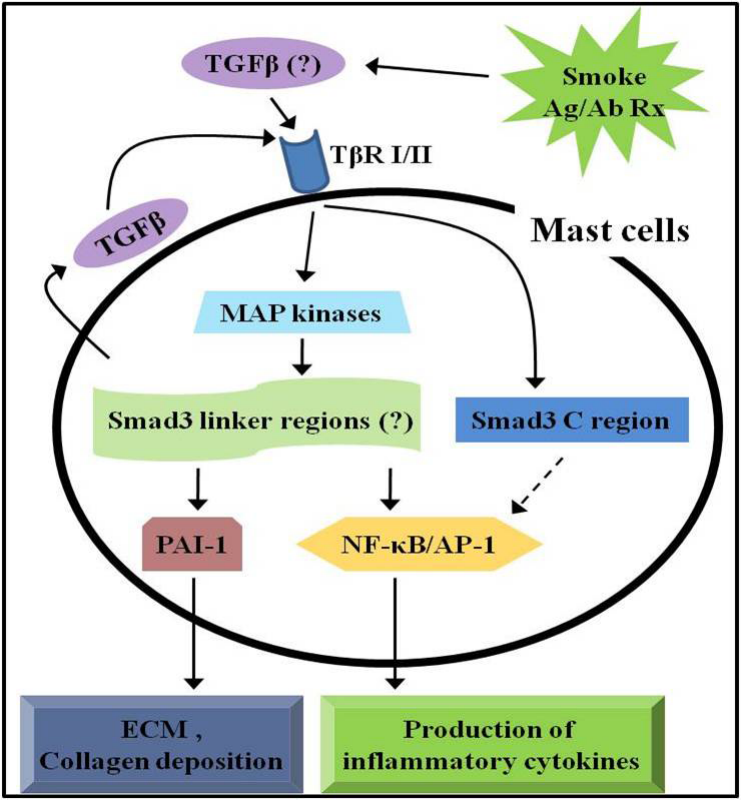
**A schematic diagram of signaling pathway in mast cells activated by antigen/antibody reaction and smoke exposure in vivo and in vitro**. The data suggest that smoke exposure induces TGF-β release from a variety of cells including mast cells. The released TGF-β activates mast cells through expression of Smad isoforms such as TβRI/pSmad3C and pSamd3L and subsequent activation of these proteins via MAP kinases. In this way, it exacerbates allergic asthma symptoms. Question mark (?) indicates TGF-β predicted to be produced by various cell types or indicates predicted signaling cascade. The dotted arrow indicates a response that was not determined in this study.

From our data, we can infer that the smoke-activated mast cells produce TGF-β, which stimulates cell activation in an autocrine manner, or TGF-β released from other cells activated by cigarette smoke may activate mast cells (Figure 8). This inference was confirmed by inhibitors of Lyn and Syk kinases, which are down-stream kinases in FcεRI signaling pathways, and by TGF-β receptor kinase inhibitor. This inference was also supported by our observation that expression of Smad proteins (a maximum expression at 6 h) occurred later than TGF-β expression in BMMCs activation (a maximum expression at 3 h). However, in vivo, TGF-β is produced not only by mast cells, but also by many other cell types including T cells [18] and eosinophils [23]. This issue will require further study to better understand the contributions of these other TGF-β-producing cells.

In general, TGF-β activates TGF-β type I receptor (TβRI) leading to direct phosphorylation of Smad3, which transduces the TGF-β signal from the cell surface to the nucleus, altering gene expression [46]. TGF-β signaling pathways include the TβRI/C-terminal phosphorylated Smad3 (pSmad3C) pathway and the MAP kinase-dependent linker phosphorylated Smad3 (pSmad3L) pathway. The pSmad3C is involved in cell growth inhibition, and pSmad3L regulates invasive capacity and extracellular matrix deposition [38,39]. As Smad3 activity was completely inhibited by MAP kinase inhibitors, and activities of MAP kinases were increased in CSE-treated/activated-BMMCs regardless Smad3 siRNA transfection, our finding is consistent with previous reports that MAP kinases exist to upstream of Smad3 in other cells (pSmad3L pathway) [38,39].

Cigarette smoke induces NF-κB and AP-1 in a variety of cells [2,3,47], and it up-regulates the expressions of inflammatory cytokines [3,4,41] and extracellular molecules [48]. Moreover, cytokine genes are regulated by NF-κB and AP-1. Therefore, our data can be inferred that smoke-activated mast cells produce TGF-β, and mast cells activated with TGF-β enhance expressions of cytokines *via *pSamd3L/NF-κB and AP-1 in the OVA-induced allergic responses (Figure 8).

Our finding for PAI-1 expression induced by smoke exposure also supports previous reports that a complex of pSmad3L associated with Smad4 undergoes translocation into the nucleus, where it binds to the Smad-binding element of the PAI-1 promoter, and induces its transcriptional activity, leading to ECM deposition [49]. The induction of PAI-1 expression is related to collagen deposition in murine asthma model [50]. In view of the fact that Smad3 siRNA transfection did not induce the activities of NF-κB or PAI-1 in Smad3 downstream signal cascades in BMMCs, we suggest that cigarette smoke may induce collagen deposition by mast cell activation through TGF-β/Smad signaling pathways.

Exposure to cigarette smoke alone (PBS/S mice) also showed similar results seen in OVA/NS mice. Therefore, it can be inferred that cigarette smoke exposure alone may induce airway inflammation and tissue remodeling.

Carbon monoxide (CO)-containing smoke is related to allergic responses to OVA [4,9] whereas other study shows no relation [11]. Melgert et al. [9] reported that smoke-exposed OVA mice, which have high levels of carcoxyhemoglobin (COHb) in serum (22.5 ± 1.7%) as compared to human smokers (4 ~ 10% COHb levels), reduced airway inflammatory cells in BAL fluid, compared to OVA mice (air instead of smoke). In our data, COHb levels (23.2 ± 1.61%) in the serum of smoking mice were similar to that of Melgert et al. [9]. However, our data showed enhancement of allergic airway responses in OVA/S mice. Therefore, we think that CO was not a significant contributor to allergic responses. However, it needs further study to better understand the relation of CO in allergic responses.

It is difficult to compare the data stimulated in vivo by whole body main stream cigarette smoke with the data from BMMCs in vitro stimulated by CSE solution. And, TGF-β production and activations of Smad3/MAP kinases are induced by more cells than by just mast cells. Therefore, our data do not support that only mast cells play an important role in smoke-exposed allergic asthma. However, as mast cells normally reside close to epithelium and blood vessels in the airway, near smooth muscle and mucus-producing gland, mast cells will be exposed and activated by antigen and smoke faster than other cells. And, we demonstrated numbers of mast cells enhanced in BAL fluid and lung tissues, correlation of the increased mast cells to smoke exposure, co-localization of mast cell tryptase and Smad3 in lung tissues, and inhibition of activities of signal molecules in BMMCs by Smad3 siRNA transfection. Therefore, we suggest that mast cells may be one of important effector cells in mouse allergic asthma exacerbated with smoke exposure, but it needs further study. Extrapolation of our data to human beings needs more study on further mechanism in animal model.

## Conclusions

The present study suggests that cigarette smoke exposure in part up-regulates antigen-induced mast cell activation associated with allergic asthma through TGF-β/pSmadL/NF-κB and AP-1 signaling pathway, and up-regulated mast cells induce the production of cytokines and collagen deposition, and then that it may exacerbate airway inflammation and tissue remodeling in mouse allergic asthma.

## List of abbreviations

OVA: ovalbumin; TGFβ: transforming growth factor-1, CSE: cigarette smoke extract; BMMCs: mouse bone marrow-derived mast cells; BAL: bronchoalveolar lavage; anti-DNP-IgE: anti-DNP (dinitrophenyl) immunoglobulin E antibody; PAI-1: plasminogen activator inhibitor type 1; MAP kinases: mitogen-activated protein kinases; ERK: extracellular signal-regulated kinase; JNK: c-jun N-terminal kinase.

## Competing interests

The authors declare that they have no competing interests.

## Authors' contributions

JYR - design of experiments, interpretation of results, analysis of the results, writing and input in terms of discussion. DYK - carrying out all BMMCs experiments. EYK - carrying out all animal experiments. GUH - smoke exposure. YSL - interpretation of results. SHL - AHR measurement and interpretation of results. All authors read and approved the final manuscript.

## Supplementary Material

Additional file 1 **Figure S1: Cell viability of BMMCs by MTT assay after stimulation with CSE**. Optimal concentration and time of CSE solution used for BMMCs stimulation were 1.0% and 6 h, respectively. Figure S2: Correlations between inflammatory cells, mast cells and goblet cells in OVA/NS and OVA/S exposed mice. Linear regression analysis for relationship between OVA/NS and OVA/S mice showed a significant relation with each r^2^. The best fit lines represent the 95% of confidence of data. Figure S3: Effects of smoke exposure on the mean linear intercept (Lm), and tidal volume and breathing frequency in lung tissues of OVA-challenged asthmatic mice. Lm values, which are shown as the sum of the length of all counting lines divided by the total number of counted intercepts, and respiratory functions including tidal volume and breathing frequency measured at 48 h after last challenge were affected by smoke exposure. Figure S4: Effects of smoke exposure, Lyn and Syk kinase inhibitors, or TGF-β receptor kinase inhibitor on the expressions and activity of Smads in BMMCs activated with antigen/antibody reaction. Protein or mRNA expressions and phosphorylation of Smads enhanced by CSE-treated/activated-BMMCs were inhibited by TGF-β receptor kinase inhibitor (SB431542), but not inhibited by Lyn (PP2) or Syk (piceatenol) inhibitor. Figure S5: Effect of smoke exposure or MAP kinase inhibitors on activity of NF-κB, AP-1 or PAI-1 in BMMCs activated with antigen/antibody reaction. The enhancement of NF-κB and AP-1 or PAI-1 activity caused by CSE-treated/activated-BMMCs was reduced by inhibitors of MAP kinases. Figure S6: Effect of smoke exposure on expressions of cytokines in BAL cells and lung tissues of OVA-induced asthmatic mice or in BMMCs activated with Ag/Ab reaction. Expression of various cytokines was enhanced in BAL cells and lung tissues or CSE-treated/activated-BMMCs more than that of OVA/NS or activated-BMMCs. Click here for file
