# Supplementary material for: Cigarette smoke exacerbates mouse allergic asthma through Smad proteins expressed in mast cells
Source: Respir Res. 2011 Apr 18;12(1):49. doi: 10.1186/1465-9921-12-49 (PMC3098800; doi:10.1186/1465-9921-12-49)
Supplement: Additional file 1 — Figure S1: Cell viability of BMMCs by MTT assay after stimulation with CSE. Optimal concentration and time of CSE solution used for BMMCs stimulation were 1.0% and 6 h, respectively. Figure S2: Correlations between inflammatory cells, mast cells and goblet cells in OVA/NS and OVA/S exposed mice. Linear regression analysis for relationship between OVA/NS and OVA/S mice showed a significant relation with each r2. The best fit lines represent the 95% of confidence of data. Figure S3: Effects of smoke exposure on the mean linear intercept (Lm), and tidal volume and breathing frequency in lung tissues of OVA-challenged asthmatic mice. Lm values, which are shown as the sum of the length of all counting lines divided by the total number of counted intercepts, and respiratory functions including tidal volume and breathing frequency measured at 48 h after last challenge were affected by smoke exposure. Figure S4: Effects of smoke exposure, Lyn and Syk kinase inhibitors, or TGF-β receptor kinase inhibitor on the expressions and activity of Smads in BMMCs activated with antigen/antibody reaction. Protein or mRNA expressions and phosphorylation of Smads enhanced by CSE-treated/activated-BMMCs were inhibited by TGF-β receptor kinase inhibitor (SB431542), but not inhibited by Lyn (PP2) or Syk (piceatenol) inhibitor. Figure S5: Effect of smoke exposure or MAP kinase inhibitors on activity of NF-κB, AP-1 or PAI-1 in BMMCs activated with antigen/antibody reaction. The enhancement of NF-κB and AP-1 or PAI-1 activity caused by CSE-treated/activated-BMMCs was reduced by inhibitors of MAP kinases. Figure S6: Effect of smoke exposure on expressions of cytokines in BAL cells and lung tissues of OVA-induced asthmatic mice or in BMMCs activated with Ag/Ab reaction. Expression of various cytokines was enhanced in BAL cells and lung tissues or CSE-treated/activated-BMMCs more than that of OVA/NS or activated-BMMCs. [file 1465-9921-12-49-S1.PDF]

Cigarette smoke exacerbates mouse allergic asthma through Smad proteins expressed in mast cells[1]

Dae Yong Kim[1,+], Eun Young Kwon[1,+], Gwan Ui Hong[1], Yun Song Lee[1], Seung-Hyo Lee[2], Jai Youl Ro[1,*]

[+]These authors equally contributed to this work

Additional File

MATERIALS

Measurement of OVA-specific serum IgE

Blood samples were collected by cardiac puncture after opening the thoracic cavity, immediately before bronchoalveolar lavage (BAL) was performed. Blood was allowed to clot at room temperature, and then centrifuged at 400 × g for 30 min. Aliquots of serum were stored at -70 °C until analyzed for OVA-specific serum IgE by ELISA.

For OVA-specific IgE, microplates were coated with purified rat anti-mouse IgE, and then treated with mouse sera followed by biotinylated OVA prepared using a peroxidase-conjugated goat anti-biotin antibody, which was used as secondary antibody. Reactions were read using an ELISA plate reader at 415 nm. Mouse IgE was used for calibrating the assay [31].

BAL fluid

Immediately after anesthesia, thoracic cavities were carefully opened. Tracheas were exposed, and BAL fluid was collected by cannulating the upper part of the trachea, by lavage twice with 1ml and then 0.8 ml of PBS (85 - 90 % of the volume inputted was recovered). Lavaged sample from each mouse were kept on ice until required. BAL fluid was centrifuged at 400 × g for 5 min, and cells were separated. After centrifugation, pellets were resuspended in 100 µl PBS, and total viable cell numbers were counted by trypan blue exclusion using a hemacytometer. BAL cells were adjusted to a concentration of 5×10^4 cells/ml in PBS. For cytospin preparations, cells were centrifuged at 400 x g for 3 min using a Cytospin III (Shandon, Pittsburg, PA), and were stained with Diff-Quik. Differential cell counting was performed using standard

morphological criteria [31].

May Grünwald-Giemsa staining

BMMCs (1×10^6 cells) were collected onto object glasses by cytospin (400 × g, 3 min). Cells were fixed in methanol for 2-3 min and then stained with May Grünwald solution for 15 min, followed by Giemsa solution for 10 min and by washing steps in H_2O [32].

Lung histology

After BAL had been performed, lungs were perfused with 5 ml PBS into the left ventricle. Exsanguinated left lungs were removed by dissection and fixed in 4% paraformaldehyde. Lung tissues embedded in paraffin were cut at 3 μm. Tissue sections were then stained with hematoxylin and eosin (H&E) for general morphology and Lm values, with periodic acid-Schiff (PAS) for the identification of goblet cells [31], and with May Grünwald-Giemsa for identification of mast cells [32]. The numbers of goblet cell hyperplasia were quantified in percentage of PAS-positive cells/total cells in 10 sites of 100 × 100 μm areas under microscopy. Right lungs were stored at -70 °C for mRNA and protein expression measurements.

Collagen deposition and amounts in lung tissues

Tissue sections were stained with Van Gieson stain for collagen deposition and examined using conventional and polarized light microscopy (Axiophot, Carl Zeiss, Oberkochen, Germany) [33]. And, collagen amounts were assessed by the Sircol collagen assay, according to the manufacturers' instruction [34]. Briefly, lung tissues

(100 mg) were homogenized and mixed with 1 ml of Sircol Dye Reagent for 30 minutes at room temperature using a mechanical shaker. The collagen-dye complex was precipitated by centrifugation at 10,000 × g for 10 minutes. The unbound dye solution was then carefully removed. The precipitated complex was resolved in 1 ml of 0.5 M sodium hydroxide. The obtained solution was placed in a 96-well flat-bottomed plate and evaluated at 550 nm using a plate reader. Absolute collagen content was calculated by comparing the samples values to a standard curve.

Measurement of mean linear intercept (L_m)

The L_m represents the average size of alveoli. The H & E-stained section was subjected to Lm analysis according to reported methods [S1]. To measure the Lm, a transparent sheet with 10 horizontal and 11 vertical lines was laid over the images. The intercepts of alveolar walls with these lines were counted. Intercepts of bronchioli or septae were counted for one half since they are more or less part of the structure of surrounding alveolar spaces. Images with bronchi, large bronchiole or blood vessels were excluded from the measurements. Images showing compression of alveolar space - observed as meandering walls - were also excluded. The Lm for each group was calculated as the sum of the length of all counting lines divided by the total number of counted intercept.

Immunohistochemistry (IHC)

Section prepared in “Lung histology” were deparaffined with xylene and washed in ethanol. Endogeneous peroxidase activity was blocked with 3 % hydrogen peroxide in methanol for 5 min. The sections were incubated for 30 min in a blocking buffer [PBS (pH 7.4), 1% BSA, 2% FCS] and incubated overnight at 4 °C with primary

rabbit anti-Smad3 or mouse anti-tryptase diluted 1:100 in PBS. After 3 × 5 min washings in PBS, the sections were incubated for 1 h with FITC-coupled goat anti-rabbit and Texas Red-coupled goat anti-mouse diluted 1:200 in PBS. Sections were then washed and examined under Confocal microscopy (LSM 5 Exciter, Carl Zeiss, Oberkochen, Germany) [35]. The degree of IHC color (yellow) developed by co-localization was quantified by intensity in 100 × 100 μm areas under microscopy (5 areas/each slide x 8 mice/each group = 40 areas), and then mean ± SE for 40 areas was presented by histogram.

Culture of Bone marrow-derived mast cells (BMMCs)

Bone marrow cells were flushed from femurs and tibias of BALB/c mice (female, 8 wk old). RBC was lysed using 0.1 M NH_4Cl and remaining cells were washed, resuspended, and cultured for 5 weeks in RPMI-1640 supplemented with 10% heat-inactivated FBS and 50% WEHI-3B conditioned media which contains IL-3. The media were changed twice a week. BMMCs (1×10^6 cells) were confirmed by May Grünwald-Giemsa staining [32].

MTT assay

The BMMCs (1×10^5 cells/well) were seeded on 96-well plates in serum free RPMI-1640 medium and 0.5% WEHI conditioned media, and final concentration 0.1, 0.5, 1.0, 1.5, 2.0, 5.0, 10.0 % of cigarette smoke extract (CSE) solution was added to each well. After incubation for 24 h, each well was added and incubated with 0.5 mg/ml MTT [3-(4,5-Dimethylthiazol-2-yl)-2,5-diphenyltetrazolium bromide] for 4 h at 37 ℃. The reaction was terminated by adding 100 μl solubilizing solution (10% SDS in 0.01M

HCl). The amount of MTT formazon product was determined at 550 nm absorbance using a spectrophotometer [S2]. The CSE solution was used in the stimulation of BMMCs at final concentration of 0.1, 0.5, and 1.0 % [36]. Optimal concentration and incubation time of CSE solution for BMMCs stimulation found in preliminary experiments were 1.0% and 6 h, respectively.

Immunoblotting for proteins

BAL cells (1 × 10^6 cells), lung tissues (50 mg) or BMMCs (1 × 10^6 cells) were homogenized in lysis buffer [10 mM Hepes (pH 7.9), 10 mM KCl, 0.1 mM EDTA, 0.1 mM EGTA, 1 mM DTT, 0.5 mM PMSF, 2.0 μg/ml aprotinin, 2.0 μg/ml leupeptin] using a Polytron (Kinematica, Littau, Switzerland), and allowed to swell on ice for 10 min. Cell lysates (μg) were subjected to 8% or 10% SDS-PAGE and transferred to nitrocellulose membranes. Membranes were washed with PBS containing 0.1% Tween 20 (PBST), and then blocked for 1 h in PBST containing 5% skim milk. After the membranes were washed with PBST, they were incubated for 60 min at room temperature with antibodies against Smad2 and 3, ERK, JNK, p38, and PAI-1, as well as p-Smad2 and 3, p-ERK, p-JNK, p-p38, diluted with PBST (1:1,000). Membranes were washed with PBST and treated with HRP-conjugated rabbit anti-goat IgG (diluted to 1:5,000 ~ 1:10,000) in PBST for 60 min. After washing, the protein bands were visualized using enhanced chemiluminescent (ECL) solution (Amersham Biosciences, Buckinghamshire, UK) [31].

Reverse transcriptase-polymerase chain reaction (RT- PCR)

Total cellular RNA was isolated from BAL cells (1 × 10^6 cells), lung tissues (50

mg) or BMMCs (1×10^6 cells) using Trizol reagent. RT-PCR was performed in a final volume of 50 μl using an amfiRivert one-step RT-PCR kit in an automated thermal cycler (BIOER Technology, Hangzhou, China). PCR assays were performed for 35 cycles. Each cycle consisted of the following steps: denaturation at 94 ℃ for 30 sec, annealing at 56 ℃ for 45 sec, and extension at 72 ℃ for 1 min. PCR products were analyzed using 1.0% agarose gel containing ethidium bromide (EtBr).

The primer sequences used were as follows: TFG-β sense, 5-CTC TCC ACC TGC AAG ACC AT-3; TFG-β anti-sense, 5-CTG CCG TAC AAT TCC AGT GA-3; Smad2 sense, 5-GCC ACA TGT TAT ATA TTG CC-3; Smad2 anti-sense, 5-TTA CAC ACT GTT GCA GGG T-3; Smad3 sense, 5-GGG CTC CCT CAT GTC ATC TA-3; Smad3 anti-sense, 5-GGC TCG CAG TAG GTA ACT GG-3; Smad4 sense, 5-AAA TGG ACA ATA TGT CTA TTA CGA ATA C-3; Smad4 anti-sense, 5-TCA GTC TAA AGG TTG TGG GTC TGC-3; Smad6 sense, 5-ATG TTC AGG TCT AAA CGT TC-3; Smad6 anti-sense, 5-CAG CGA GTA CGT GAC CG-3; Smad7 sense, 5-CTC CTG CTG TGC AAA TGT TTC-3; Smad7 anti-sense, 5-CAG GCT CCA GAA GAA GTT GG-3; IL-1β sense, 5-TGA AGG GCT GCT TCC AAA CCT TTG ACC-3; IL-1β anti-sense, 5-TGT CCA TTG AGG TGG AGA GCT TTC AGG-3; IL-4 sense, 5-TCG GCA TTT TGA ACG AGG TC-3; IL-4 anti-sense, 5-GAA AAG CCC GAA AGA GTC TC-3; IL-5 sense, 5-ATG GAG ATT CCC ATG AGC AC-3; IL-5 anti-sense, 5-GTC TCT CCT CGC CAC ACT TC-3; IL-6 sense, 5-TGG AGT CAC AGA AGG AGT GGC TAA G-3-3; IL-6 anti-sense, 5-TCT GAC CAC AGT GAG GAA TGT CCA C-3; IL-10 sense, 5-CAT GGG TCT TGG GAA GAG AA-3; IL-10 anti-sense, CAT TCC CAG AGG AAT TGC AT-3; IL-13 sense, 5-CAG CTC CCT GGT TCT CTC AC-3; IL-13 anti-sense, 5-CCA CAC TCC ATA CCA TGC TG-3; TNFα sense, 5-TTA TCT CTC AGC

TCC ACG CC-3; TNFα anti-sense, 5-TGC GCA CTG AAA GCA TGA TC-3; INF-γ sense, 5-GCT CTG AGA CAA TGA ACG CT-3; IFN-γ anti-sense, 5-AAA GAG ATA ATC TGG CTG TGC-3; GAPDH sense, 5-GAT GCA GGG ATG ATG TTC TG-3; GAPDH anti-sense, 5-GTG AAG GTC GGT AAC GG-3 [31].

Enzyme linked immunosorbent assay (ELISA)

The levels of IL-4, IL-5, IL-13, TNF-α and TGF-β (total and active forms) levels in supernatants isolated from lung tissue homogenates or in media of activated-BMMCs were determined by ELISA. Lung tissues (1 mg/500 μl) were homogenized in PBS using a Polytron (Kinematica, Littau, Switzerland) and centrifuged (20,000 x g, 10 min); supernatants were collected. And, supernatants were also collected from media of BMMCs (1×10^6 cells) activated with anti-DNP-IgE antibody/DNP-HSA. For active TGF-β1 detection, supernatants were added 1 N HCl, incubated 10 min at room temperature, and then neutralized the acidified sample by adding 13 μl of 1.2 N NaOH/0.5 M Hepes. Ninety-six well plates were coated with each of the cytokine antibodies (i.e., anti-TFG-β, IL-4, IL-5, TNF-α; anti-IL-13) overnight at 4 °C. Plates were blocked with PBS containing 10 % FBS for 1 h at room temperature, and incubated with 100 μl of standard, the supernatants of lung homogenates or BMMCs for 2 h at room temperature. One hundred microliters of biotinylated anti-TGF-β, IL-4, IL-5, TNF-α, IL-13 and avidin-horseradish peroxidase conjugate were then added. After washing, substrate solution (tetramethylbenzidine and hydrogen peroxide) was added to each well, and optical density was read at 450 nm. Standard curves were generated using different concentrations of each of the recombinant cytokines. The limit of

detection of this method was better than 7.8 pg/ml.

Electrophoretic mobility shift assay (EMSA)

To prepare nuclear extracts, BMMCs (1 × 10^6 cells) were washed twice with ice-cold PBS and resuspended in 1 ml ice-cold buffer A [10 mM Hepes/KOH (pH 7.9), 10 mM KCl, 1.5 mM $MgCl_2$, 0.5 mM DTT, 0.2 mM PMSF, 1μg/ml leupeptin, and 1 μg/ml aprotinin]. After incubation on ice for 15 min, the cells were lysed by adding Nonidet P40 (10 μl 10% Nonidet P40, to a final concentration of 0.625 %, v/v) and immediately vortexed for 10 sec. Nuclei were harvested by centrifugation at 20,000 x g for 1 min and resuspended in 40 μl ice-cold buffer C [20 mM Hepes/KOH (pH 7.9), 0.42 M NaCl, 1.5 mM $MgCl_2$, 0.2 mM EDTA, 0.5 mM DTT, 25 % glycerol, 0.2 mM PMSF, 1 μg/ml leupeptin, and 1 μg/ml aprotinin]. After incubation at 4℃ for 20 min on a shaking platform, the nuclei were clarified by centrifugation at 15,000 × g for 10 min. The supernatant (nuclear extract) was then transferred to a new tube and quantified using Bradford's method. Nuclear extracts were stored at -70 ℃ until required [31].

Ten microliters of a mixture of NF-κB (5-AGT TGA GGG GAC TTT CCC AGG C-3, 3-TCA ACT CCC CTG AAA GGG TCC G-5) or AP-1 (5-CGC TTG ATG AGT CAG CCG GAA-3, 3-GCG AAC TAC TCA GTC GGC CTT-5) oligonucleotide (1.75 pmol/μl), T4 polynucleotide kinase 10X buffer, [α-^{32}P]ATP (10 μCi; 3,000 Ci/mmol), nuclease-free water, and T4 polynucleotide kinase (5 ~ 10 U/μl) were incubated at 37°C for 30 min. The reaction was stopped by adding 1 μl EDTA (0.5 M). After adding 89 μl Tris-EDTA (TE) buffer [10 mM Tris-HCl (pH 8.0), 1 mM EDTA], unincorporated nucleotides were separated from the DNA probe by G-25 spin column chromatography. The nuclear extract and gel shift binding 5X buffer [20% glycerol, 5

mM $MgCl_2$, 2.5 mM EDTA, 2.5 mM DTT, 250 mM NaCl, 50mM Tris-HCl (pH 7.5), and 0.25 mg/ml poly (dI-dC)] were incubated at room temperature for 10 min, and then 20-30 fmol of ^{32}P-labeled NF-κB or AP-1 oligonucleotide was added and incubated at room temperature for 20 min. After stopping the reaction, 1μl of 10 X gel loading buffer was added to each reaction. Reaction mixtures were electrophoresed on 6% polyacrylamide gels, and the gels were analyzed using FLA-2000 (Fujifilm, Tokyo, Japan).

REFERENCES

S1. Lee J, Reddy R, Barsky L, Scholes J, Chen H, Shi W, Driscoll B: **Lung alveolar integrity is compromised by telomere shortening in telomerase-null mice.** *Am J Physiol Lung Cell Mol Physiol* 2009, **296**:L57-L70.

S2. Eckstein N, Servan K, Girard L, Cai D, von Jonquieres G, Jaehde U, Kassack MU, Gazdar AF, Minna JD, Royer HD. **Epidermal growth factor receptor pathway analysis identifies amphiregulin as a key factor for cisplatin resistance of human breast cancer cells.** *J Biol Chem* 2008; **283**:739-750.

Legends

Figure S1. Cell viability of BMMCs by MTT assay after stimulation with CSE. BMMCs (1 × 10^5 cells/well) were seeded on 96-well plates and cultured in serum free RPMI-1640 and 0.5% WEHI conditioned media. Final concentration of CSE was 0.1, 0.5, 1.0, 1.5 or 2.0 %. After incubation for 24h, BMMCs were incubated with 0.5 mg/ml MTT [3-(4,5-Dimethylthiazol-2-yl)-2,5-diphenyltetrazolium bromide] for 4hr at 37℃. The reaction was terminated by adding 100 μl of solubilizing solution (10% SDS in 0.01M HCl) and determined at 550 nm using a spectrophotometer.

Figure S2. Correlations between inflammatory cells, mast cells and goblet cells in OVA/NS and OVA/S exposed mice. Relationship between OVA/NS and OVA/S mice was determined by linear regression analysis. Linear regression analysis shows a significant relation with each r^2. The best fit lines represent the 95% of confidence of data. Each dot represents one mouse.

Figure S3. Effects of smoke exposure on the mean linear intercept (Lm), and tidal volume and breathing frequency in lung tissues of OVA-challenged asthmatic mice. PBS/NS, mice sensitized and nebulized by PBS without smoke exposure; OVA/NS, mice sensitized and nebulized by OVA without smoke exposure; PBS/S, mice sensitized and nebulized by PBS with smoke exposure; OVA/S, mice sensitized and nebulized by OVA with smoke exposure. General sensitization and local challenge were performed 10 min after final smoke exposure. Lung tissues were fixed with 4% paraformaldehyde, sectioned, and stained with hematoxilin and eosin (H & E) for measuring Lm values (magnification: × 200). Lm values for each group were calculated

as the sum of the length of all counting lines divided by the total number of counted intercepts (A). Respiratory function was measured at 48 h after last challenge using a whole-body plethysmography as described in “Materials and Methods”. (B) Tidal volume. (C) Breathing frequency. Data are shown as mean ± SEM for each group (n = 8). *, P < 0.05; **, *P* < 0.01; ***, *P* < 0.001 versus PBS/NS mice. †, *P* < 0.05; ††, *P* < 0.01; †††, *P* < 0.001 versus OVA/NS mice. ‡, *P* < 0.05; ‡‡, *P* < 0.01 versus PBS/S mice.

Figure S4. Effects of smoke exposure, Lyn and Syk kinase inhibitors, or TGF-β receptor kinase inhibitor on the expressions and activity of Smads in BMMCs activated with antigen/antibody reaction. BMMCs (1×10^{6} cells) were sensitized with 0.1 μg/ml anti-DNP IgE antibody, stimulated with DNP-HSA (1.0 μg/ml) for 6 h, and CSE solution (OD 2.0, 1.0%) was treated during DNP-HAS stimulation, as described in “Materials and Methods”. Lyn and Syk kinase inhibitor (10 μM PP2 and 25 μM piceatenol, respectively) or TGF-β receptor kinase inhibitor (10 μM SB431542) were added 10 min and 30 min before antigen (DNP-HSA) challenge, respectively. mRNA expressions or activity of Smads were determined in nuclear protein extracts and extract protein prepared from BMMCs by RT-PCR and western blot, respectively. (A-left panel) Time course of mRNA expression of Smads; (A-right panel) Smad mRNA in CSE-treated/activated-BMMCs for 6 h. (B) mRNA expression (left panel) and activity of Smad3 (right panel) for Lyn or Syk inhibitor pretreatment. (C) Concentration responses (left panel) and time courses (right panel) in the pretreatment of TGF-β receptor kinase inhibitor (SB431542). Ag/Ab Rx, anti-DNP IgE antibody/DNP-HSA reaction; -, +, without and with substances; pice, piceatenol; BM, BMMCs. *, Numbers

below the bands indicate the ratio of band density of each group versus those of the negative control and GAPDH mRNA, tubulin or actin. Data shown are representative of four independent experiments (n = 4).

Figure S5. Effect of smoke exposure or MAP kinase inhibitors on activity of NF-κB, AP-1 or PAI-1 in BMMCs activated with antigen/antibody reaction. Experimental conditions used were described in Figure S1. MAP kinase inhibitors (50 μM PD98059 for ERK, 10 μM SP600125 for JNK and 10 μM SB205380 for p38 kinase) were added 30 min before DNP-HSA stimulation. The NF-κB (A-left panel) and AP-1 (A-right panel) activities or PAI-1 expression (B) were determined in nuclear protein extracts prepared from BMMCs using EMSA or western blot, respectively. a, negative control; b, competition assay; Ag/Ab Rx, anti-DNP IgE antibody/DNP-HSA reaction; -, +, without and with substances. *, Numbers below the bands are the ratio of band density of each group versus those of the negative control and each total protein or actin.

Figure S6. Effect of smoke exposure on expressions of cytokines in BAL cells and lung tissues of OVA-induced asthmatic mice or in BMMCs activated with Ag/Ab reaction. Experimental conditions and group symbols used in BAL cells and lung tissues or BMMCs were described in Figure S2 and Figure S3, respectively. The expressions of cytokine mRNA was determined by RT-PCR. Ag/Ab Rx, anti-DNP IgE antibody/DNP-HSA reaction. Left panel, BAL cells; middle panel, lung tissues; right panel, BMMCs; -, +, without and with substances. *, Numbers below the bands are the

ratio of band density of each group versus those of PBS/NS or the negative control and GAPDH mRNA.

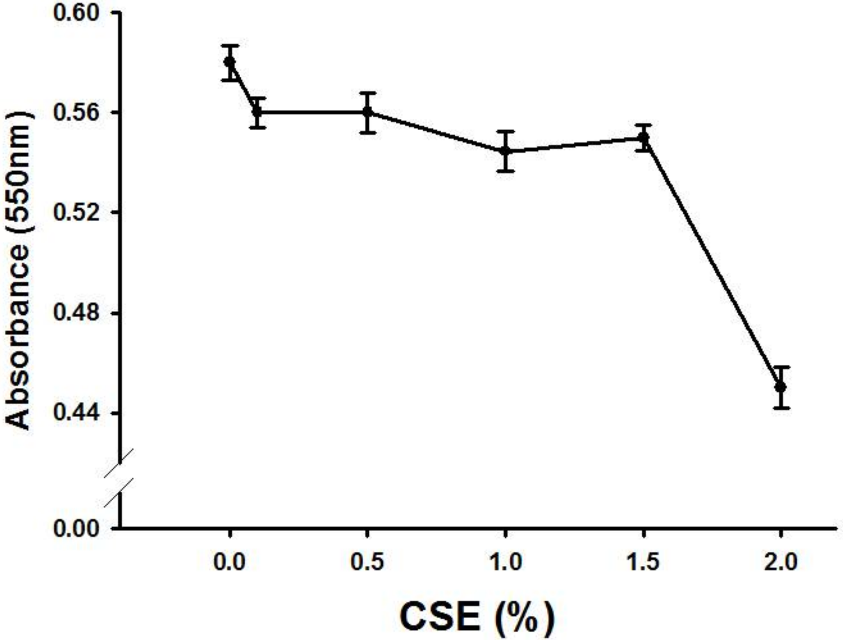

Absorbance (550nm)
0.60
0.56
0.52
0.48
0.44
0.00
0.0
0.5
1.0
1.5
2.0
CSE (%)

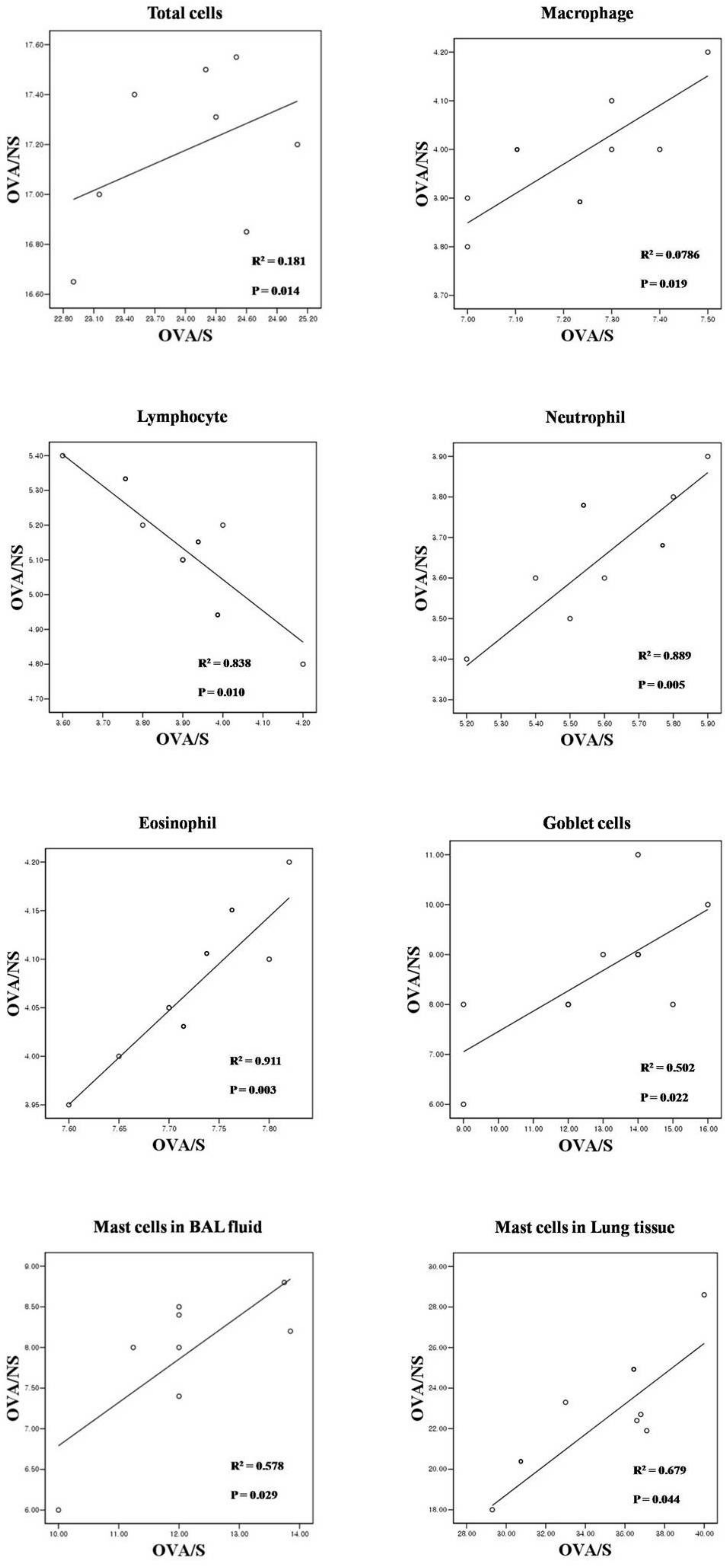
Total cells
OVA/NS
OVA/S
R² = 0.181
P = 0.014
Macrophage
OVA/NS
OVA/S
R² = 0.0786
P = 0.019
Lymphocyte
OVA/NS
OVA/S
R² = 0.838
P = 0.010
Neutrophil
OVA/NS
OVA/S
R² = 0.889
P = 0.005
Eosinophil
OVA/NS
OVA/S
R² = 0.911
P = 0.003
Goblet cells
OVA/NS
OVA/S
R² = 0.502
P = 0.022
Mast cells in BAL fluid
OVA/NS
OVA/S
R² = 0.578
P = 0.029
Mast cells in Lung tissue
OVA/NS
OVA/S
R² = 0.679
P = 0.044

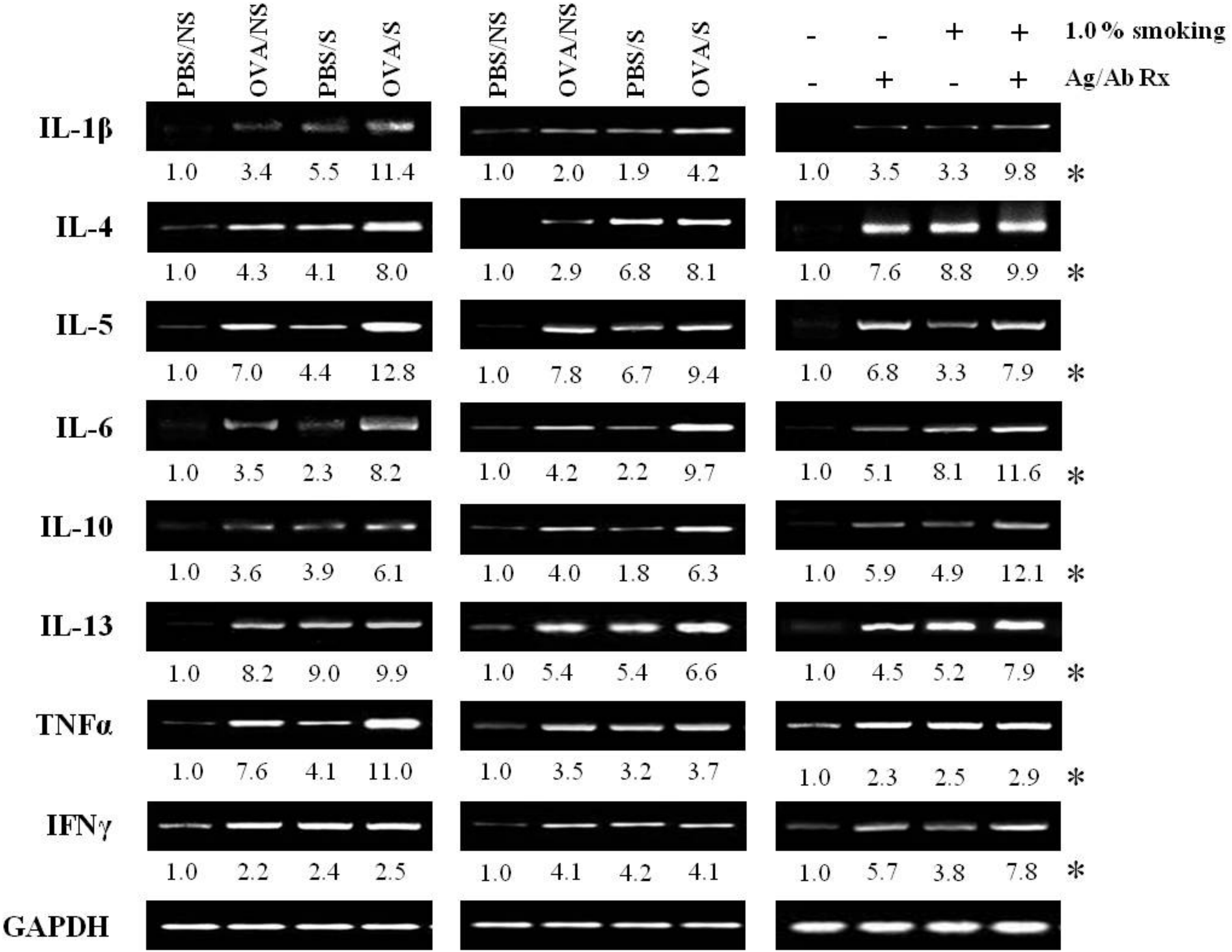

PBS/NS
OVA/NS
PBS/S
OVA/S
PBS/NS
OVA/NS
PBS/S
OVA/S
- - + + 1.0 % smoking
- + - + Ag/Ab Rx
IL-1β
1.0 3.4 5.5 11.4
1.0 2.0 1.9 4.2
1.0 3.5 3.3 9.8 *
IL-4
1.0 4.3 4.1 8.0
1.0 2.9 6.8 8.1
1.0 7.6 8.8 9.9 *
IL-5
1.0 7.0 4.4 12.8
1.0 7.8 6.7 9.4
1.0 6.8 3.3 7.9 *
IL-6
1.0 3.5 2.3 8.2
1.0 4.2 2.2 9.7
1.0 5.1 8.1 11.6 *
IL-10
1.0 3.6 3.9 6.1
1.0 4.0 1.8 6.3
1.0 5.9 4.9 12.1 *
IL-13
1.0 8.2 9.0 9.9
1.0 5.4 5.4 6.6
1.0 4.5 5.2 7.9 *
TNFα
1.0 7.6 4.1 11.0
1.0 3.5 3.2 3.7
1.0 2.3 2.5 2.9 *
IFNγ
1.0 2.2 2.4 2.5
1.0 4.1 4.2 4.1
1.0 5.7 3.8 7.8 *
GAPDH

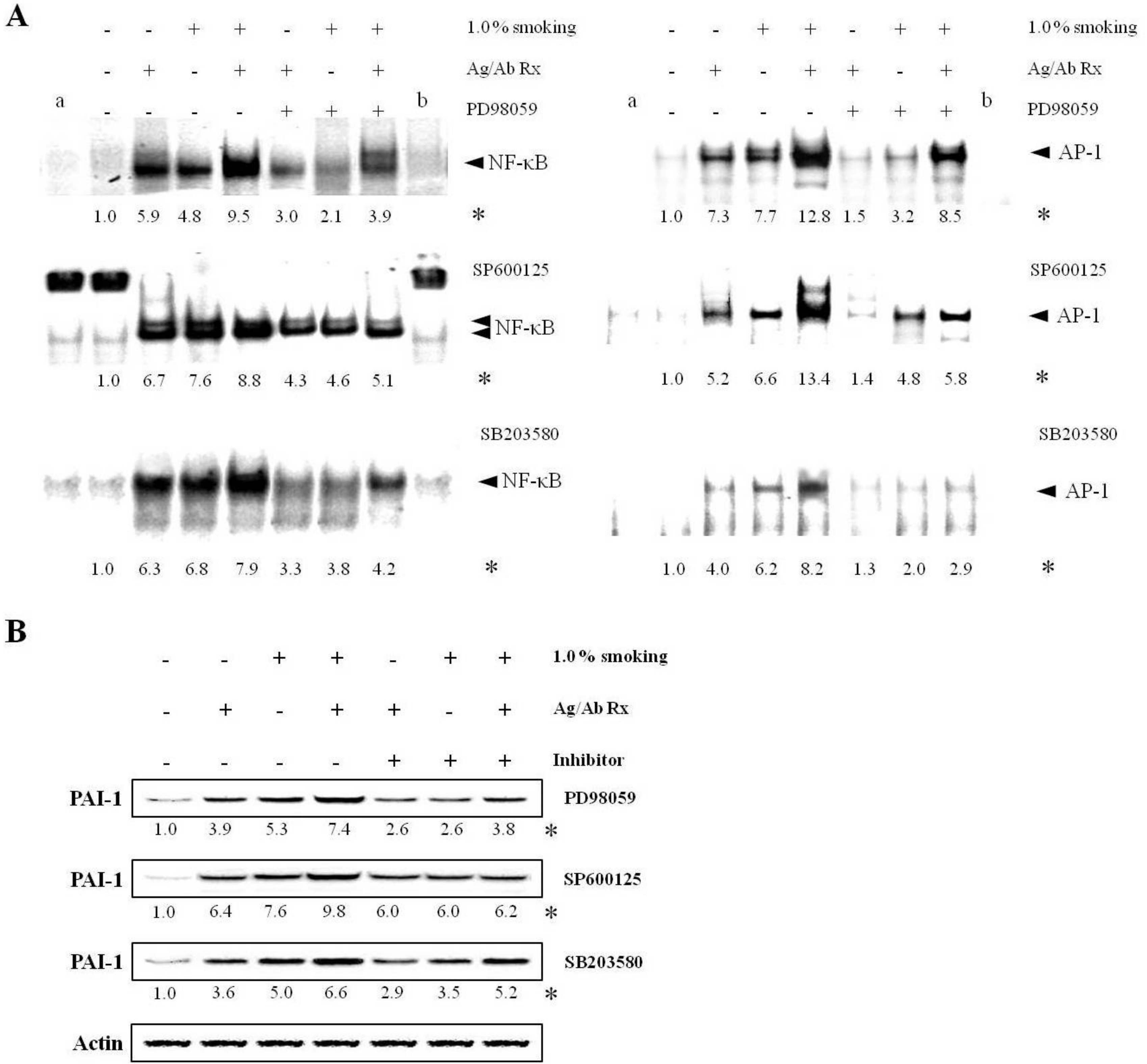
A
1.0% smoking
Ag/Ab Rx
PD98059
a
b
NF-κB
1.0 5.9 4.8 9.5 3.0 2.1 3.9 *
SP600125
NF-κB
1.0 6.7 7.6 8.8 4.3 4.6 5.1 *
SB203580
NF-κB
1.0 6.3 6.8 7.9 3.3 3.8 4.2 *
AP-1
1.0 7.3 7.7 12.8 1.5 3.2 8.5 *
AP-1
1.0 5.2 6.6 13.4 1.4 4.8 5.8 *
AP-1
1.0 4.0 6.2 8.2 1.3 2.0 2.9 *
B
1.0% smoking
Ag/Ab Rx
Inhibitor
PAI-1
PD98059
1.0 3.9 5.3 7.4 2.6 2.6 3.8 *
SP600125
1.0 6.4 7.6 9.8 6.0 6.0 6.2 *
SB203580
1.0 3.6 5.0 6.6 2.9 3.5 5.2 *
Actin

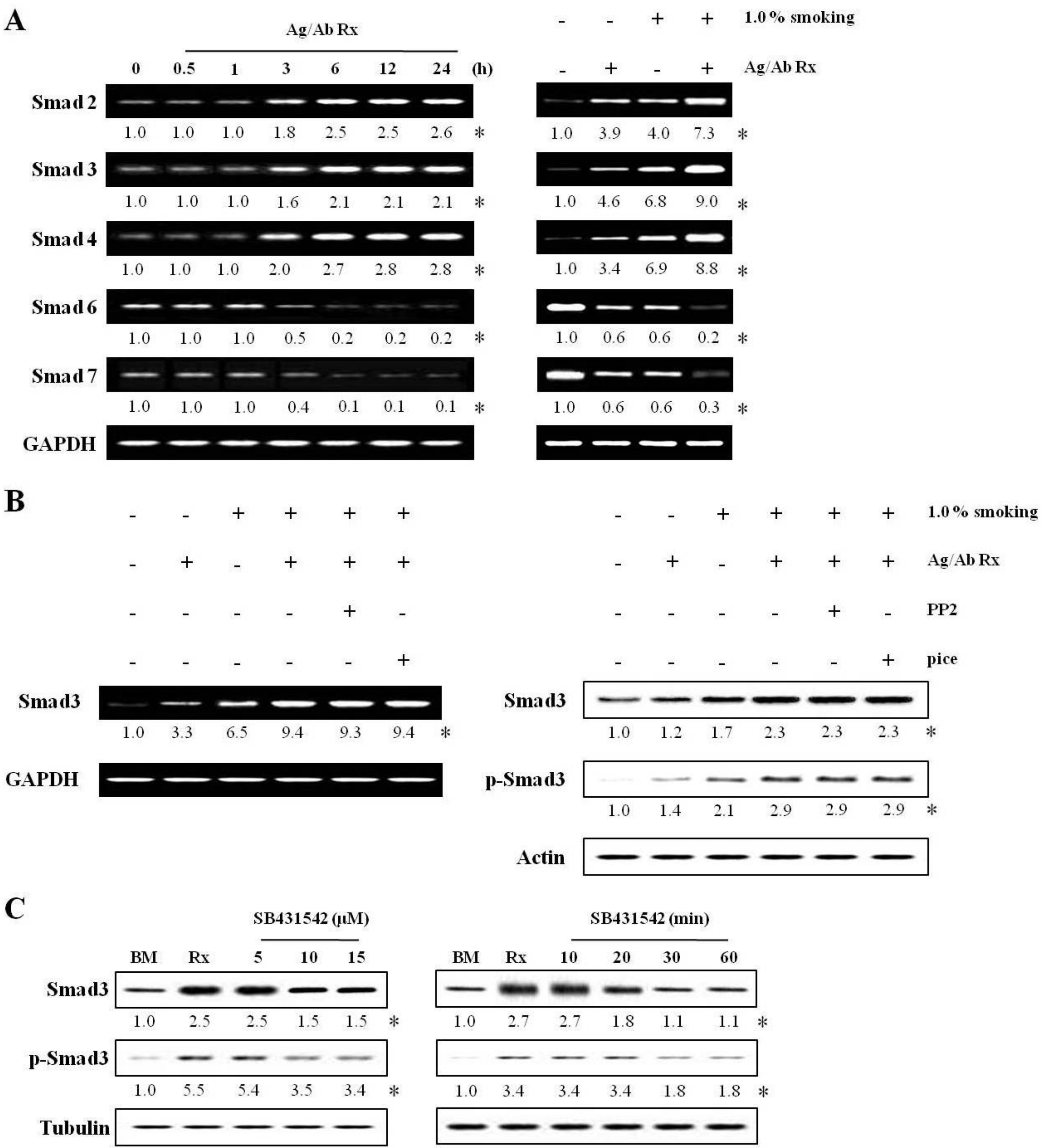

A
Ag/Ab Rx
0 0.5 1 3 6 12 24 (h)
Smad 2
1.0 1.0 1.0 1.8 2.5 2.5 2.6 *
Smad 3
1.0 1.0 1.0 1.6 2.1 2.1 2.1 *
Smad 4
1.0 1.0 1.0 2.0 2.7 2.8 2.8 *
Smad 6
1.0 1.0 1.0 0.5 0.2 0.2 0.2 *
Smad 7
1.0 1.0 1.0 0.4 0.1 0.1 0.1 *
GAPDH
- - + + 1.0 % smoking
- + - + Ag/Ab Rx
1.0 3.9 4.0 7.3 *
1.0 4.6 6.8 9.0 *
1.0 3.4 6.9 8.8 *
1.0 0.6 0.6 0.2 *
1.0 0.6 0.6 0.3 *
B
- - + + + + 1.0 % smoking
- + - + + + Ag/Ab Rx
- - - - + - PP2
- - - - - + pice
Smad3
1.0 3.3 6.5 9.4 9.3 9.4 *
GAPDH
Smad3
1.0 1.2 1.7 2.3 2.3 2.3 *
p-Smad3
1.0 1.4 2.1 2.9 2.9 2.9 *
Actin
C
SB431542 (μM)
BM Rx 5 10 15
Smad3
1.0 2.5 2.5 1.5 1.5 *
p-Smad3
1.0 5.5 5.4 3.5 3.4 *
Tubulin
SB431542 (min)
BM Rx 10 20 30 60
1.0 2.7 2.7 1.8 1.1 1.1 *
1.0 3.4 3.4 3.4 1.8 1.8 *

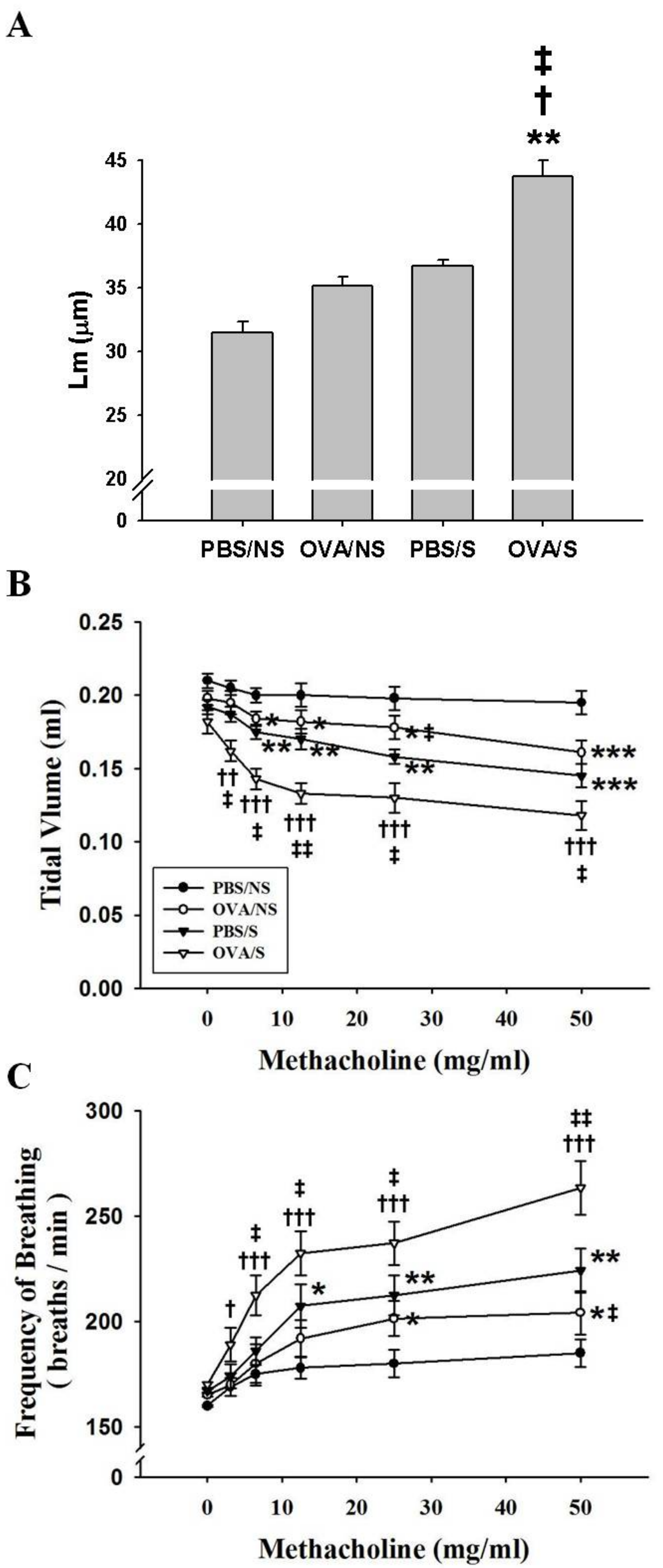
A
‡
†
**
45
40
35
30
25
20
0
Lm (μm)
PBS/NS
OVA/NS
PBS/S
OVA/S
B
0.25
0.20
0.15
0.10
0.05
0.00
Tidal Vlume (ml)
PBS/NS
OVA/NS
PBS/S
OVA/S
0
10
20
30
40
50
Methacholine (mg/ml)
C
300
250
200
150
0
Frequency of Breathing
(breaths / min)
0
10
20
30
40
50
Methacholine (mg/ml)
